# Effect of soy isoflavone supplementation on blood pressure: a meta-analysis of randomized controlled trials

**DOI:** 10.1186/s12937-024-00932-6

**Published:** 2024-03-07

**Authors:** Lifu Lei, Suocheng Hui, Yushi Chen, Hongjia Yan, Jian Yang, Shiwen Tong

**Affiliations:** 1 Department of Clinical Nutrition, The Second Affiliated Hospital of Chongqing Medical University, Chongqing, 400016 China; 2 Department of Clinical Nutrition, The People’s Hospital of Chongqing Liang Jiang New Area, Chongqing, 401135 China; 3 Department of Clinical Nutrition, The Third Affiliated Hospital of Chongqing Medical University, Chongqing, 410020 China; 4 Research Center for Metabolic and Cardiovascular Diseases, The Third Affiliated Hospital of Chongqing Medical University, Chongqing, 410020 China

**Keywords:** Soy isoflavone, Systolic blood pressure, Diastolic blood pressure, Hypertension, Dose-response

## Abstract

**Background:**

Previous experimental studies have suggested that the consumption of soy isoflavones may have a potential impact on lowering blood pressure. Nevertheless, epidemiological studies have presented conflicting outcomes concerning the correlation between soy isoflavone consumption and blood pressure levels. Consequently, a comprehensive meta-analysis of all eligible randomized controlled trials (RCTs) was conducted to explore the influence of soy isoflavones on systolic blood pressure (SBP) and diastolic blood pressure (DBP) in adults.

**Methods:**

A thorough search of PubMed, Embase, and the Cochrane Library for relevant literature up to April 30, 2023 was conducted. RCTs involving adults that compared soy isoflavone supplementation with a placebo (the same matrix devoid of soy isoflavone) were included. The combined effect size was presented as the weighted mean difference (WMD) along with 95% confidence interval (CI), employing a fixed-effects model.

**Results:**

Our meta-analysis included a total of 24 studies involving 1945 participants. The results revealed a significant reduction in both SBP and DBP with soy isoflavone supplementation. Subgroup analyses suggested more pronounced reductions in SBP and DBP for interventions lasting ≥6 months, in individuals receiving mixed-type soy isoflavone, and among patients with metabolic syndrome or prehypertension. However, we did not detect significant nonlinear associations between supplementation dosage and intervention duration concerning both SBP and DBP. The overall quality of evidence was deemed moderate.

**Conclusions:**

The current meta-analysis revealed that supplementation with soy isoflavones alone effectively reduces blood pressure. Additional high-quality studies are required to investigate the efficacy of blood pressure reduction through supplementation with an optimal quantity and proportion of soy isoflavone.

**Supplementary Information:**

The online version contains supplementary material available at 10.1186/s12937-024-00932-6.

## Introduction

Hypertension is identified as the most significant risk factor for cardiovascular diseases (CVDs) and accounts for 10.8 million deaths globally each year [1–3]. The high prevalence of hypertension is a global public health problem that is increasing the healthcare burden in many countries [3–5]. Therefore, preventing the development of hypertension and rationally controlling blood pressure are effective strategies for reducing medical consumption and improving global health [5, 6].

Lifestyle modifications, including increasing physical activity, maintaining a healthy diet, and dietary supplementation, are essential strategies for the prevention and treatment of hypertension [7]. Recent evidence has demonstrated that specific nutrient supplementation is associated with lower blood pressure [8, 9]. Soy isoflavone is a phytoestrogen originating from soybean that mainly includes genistein, daidzein, and glycitein. It has numerous physiological functions, such as anti-inflammatory, antioxidant and cardioprotective activities [10]. Moreover, soy isoflavones have been shown to be beneficial for treating several chronic diseases, including menopausal symptoms, obesity, diabetes, and CVDs, including hypertension [11, 12]. The levels of soy isoflavone and its bioactive metabolite equol are significantly negatively associated with the incidence of CHD [13–15]. A large population cohort study indicated that soy isoflavone consumption was inversely related to the risk of myocardial infarction [16]. Moreover, genistein and daidzein have been shown to exert anti-hypertensive effects in hypertensive model animals across numerous experimental studies [17–20]. Thus, the role of soy isoflavone in cardiovascular protection has attracted increasing attention.

The impact of soy isoflavone on blood pressure remains a subject of ongoing debate in epidemiological studies. Some randomized controlled trials (RCTs) have suggested potential benefits of soy isoflavone supplementation on blood pressure [21–23], while others have not observed such an influence [24–26]. A prior meta-analysis has reported that soy isoflavone supplementation leads to a reduction in systolic blood pressure (SBP), but does not affect diastolic blood pressure (DBP) [27]. Another meta-analysis has shown that soy isoflavone supplementation lowers both SBP and DBP among individuals with hypertension [28]. It is worth noting that the trials included in this study involved soy proteins, making it challenging to pinpoint which component is responsible for the blood pressure effect. Previous findings remain inconsistent, and it is unclear whether soy isoflavones have beneficial effects on blood pressure independent of soy protein. These meta-analyses were conducted more than a decade ago, and since then, multiple new RCTs have been published to provide new evidence. Considering the aforementioned points, the objective was to assess the effects of soy isoflavone on the prevention of hypertension by examining the impact of soy isoflavone supplementation alone on SBP and DBP in adults, especially in participants with varying doses of intervention, durations of intervention, and characteristics. To fulfill this aim, we conducted a comprehensive dose-response meta-analysis of all suitable trials, and we evaluated the quality of the evidence using the Grading of Recommendations Assessment, Development, and Evaluation (GRADE) approach.

## Methods

This meta-analysis was conducted according to the guidelines outlined in the Preferred Reporting Items for Systematic Reviews and Meta-Analyses statement (PRISMA) [29] and was registered with the International Prospective Register of Systematic Reviews (PROSPERO, CRD42023408560).

### Literature search strategy

A comprehensive literature search, including studies published on or before April 30, 2023, was performed using the using Pubmed (https://pubmed.ncbi.nlm.nih.gov), Embase (http://www.embase.com/search/advanced), and Cochrane Library databases (https://www.cochrane.org). Medical subject headings and keywords were employed to search for terms such as soy, soy protein, isoflavone, phytoestrogen, genistein, daidzein, glycitein, blood pressure, hypertension, antihypertensive agents, and hypotension. Two authors independently screened and evaluated titles and abstracts for each study. Subsequently, studies meeting the inclusion criteria had their full texts documented, and any discrepancies were resolved through discussion involving a third reviewer. The agreement on the systematic search among investigators was estimated using Cohen’s kappa test. The detailed search strategy is presented in Table S1.

### Selection criteria

Studies were included if they met the following inclusion criteria: (1) had a randomized controlled trial (RCT) design, including a parallel or crossover design; (2) included participants were adults (≥18 years old) in the study; (3) had an intervention duration of at least 4 weeks; (4) had an intervention group that contained soy isoflavone and a control group that had a placebo (if the intervention group had soy isoflavone and other compounds, the control group had the same compound as a placebo); (5) had SBP or/and DBP reported as primary or secondary outcomes; (6) had mean/median values for SBP or DBP with standard errors of the means (SEM) or standard deviations (SD) or 95% confidence interval (Cl) for the control and soy isoflavone groups. (7) were reported in the English language.

Studies were excluded if they met the following criteria: (1) were observational studies, non-clinical trials, or animal studies; (2) were children, adolescents, pregnant or lactating women, or individuals with preexisting cardiovascular events (e.g., stroke or heart failure), renal diseases, or secondary hypertension.

### Data extraction

Data from each included study was independently extracted by two investigators (L. Lei and S. Hui): (1) Study characteristics, including the first author’s name, publication year, country, intervention duration, study design type, sample size (for soy isoflavone and control groups), average age, body mass index (BMI), gender distribution (percentage of women), subjects’ health status, intervention details in the soy isoflavone and control groups, and results. (2) Data for the study endpoints were recorded when outcomes were available at various time points in the studies.

### Quality assessment

The risk of bias in the eligible studies was assessed by two investigators using the Cochrane Collaboration’s tool [30], which included the following six aspects: (1) selection bias; (2) performance bias; (3) detection bias; (4) attrition bias; (5) reporting bias; (6) other bias, including baseline comparisons. Studies were categorized as high risk if they exhibited one or more items with a high risk of bias, and as low risk if all items had low bias risk. Other studies were evaluated as having an unclear risk of bias. Furthermore, the quality of each outcome was graded using GRADE, which included criteria such as study design, risk of bias, imprecision, inconsistency, indirectness, and publication bias. The level of evidence was assessed as high, moderate, low, or very low [31, 32]. Two investigators (L. Lei and S. Hui) independently evaluated study quality (risk of bias and GRADE), with discrepancies resolved through consensus or referral to a third author (S. Tong).

### Statistical analysis

The weighted mean difference (WMD) between the soy isoflavone and control groups was calculated using the mean change and SD of SBP and DBP measurements. The baseline and outcome values can be found in Table 2 of Supplementary Material 3. In cases where the SD of the mean change was not reported in the studies, we estimated it using the following formula: SD = square root [(SD_baseline_^2^ + SD_endpoint_^2^) - (2 × R × SD_baseline_ × SD_endpoint_)], assuming a correlation coefficient of 0.5 [33]. When the SEM of the mean change was provided in the trials, we calculated SD as follows: SD = SEM × square root (n), where n was the number of participants. For cases where 95% CI values were reported, the method of calculating SD was described by Hozo *et al.* [34].

To assess the heterogeneity of the included studies, we used the Higgins index (*I*^*2*^) and *P* value [35]. If significant heterogeneity was present (*I*^2^ >50% or *P* <0.1), the random effects model was utilized; otherwise, the fixed effects model was applied. Publication bias was assessed through visual inspection of funnel plots and statistical testing using Egger’s test [36]. To explore potential sources of heterogeneity, subgroup analyses were conducted based on factors such as soy isoflavone dosage, intervention duration, baseline BMI, gender, mean age, resting blood pressure status (normotension: <120 mmHg SBP and <80 mmHg DBP; prehypertension: 120-139 mmHg SBP and/or 80-89 mmHg DBP; hypertension: ≥140 mmHg SBP and/or ≥90 mmHg DBP) [37–39], participants’ health status, and types of soy isoflavone. Meta-regression was conducted to examine the relationship between the effect size and several moderators, such as the dosage and duration of intervention. Non-linear effects of soy isoflavone dosage (mg/day) and intervention duration (months) were explored using fractional polynomial modeling (polynomials) [40]. The robustness of the studies was assessed through sensitivity analyses, which involved excluding each study one by one and conducting the analysis [41]. All statistical analyses were carried out using Stata statistical software (Version 14.0; Stata Corp.), and statistical significance was determined at* P* values <0.05.

## Results

### Results of literature search

**Fig. 1:**
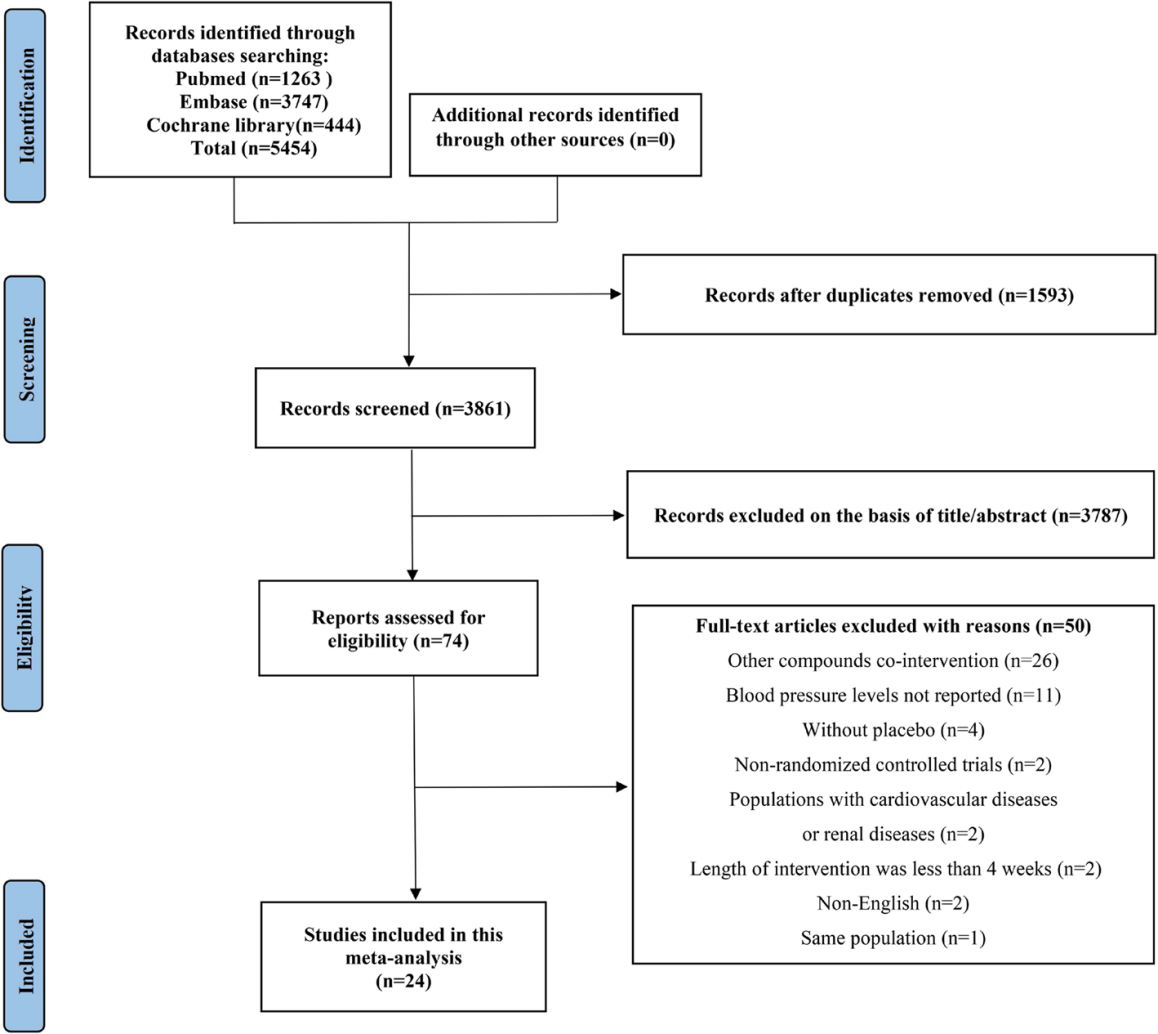
Flow diagram of study selection for meta-analysis.

The study selection procedure for this meta-analysis is illustrated in Fig. 1. The inclusion and exclusion of articles were in substantial agreement among the investigators (Kappa = 0.86; % agreement = 98.56; *P* <0.001). Initially, a total of 5454 relevant studies were identified through the search, and subsequently, 1593 duplicate studies were eliminated. After reviewing the titles and/or abstracts, 3787 studies were further excluded. Furthermore, upon a comprehensive evaluation of the full texts, 50 studies were excluded as they did not meet the inclusion criteria. Ultimately, 24 studies satisfied the inclusion criteria and were included in this meta-analysis.

### Characteristics of included studies

The essential characteristics of the 24 studies included in this meta-analysis are summarized in Table 1 of Supplementary Material 3 [21–26, 42–59]. The study included a total of 1945 participants, whose ages ranged from 44 to 74 years at baseline. Among them, 1005 individuals were part of the soy isoflavone group, while 940 participants belonged to the control group. The participants consisted of healthy individuals and patients with conditions such as type 2 diabetes, metabolic syndrome, insulin resistance, and non-alcoholic fatty liver disease. Two studies included both males and females, one study exclusively had male participants, and the remaining twenty-one studies involved only female participants. The baseline BMI of the participants varied from 23 to 32 kg/m^2^. These studies were published between 2000 and 2018. The intervention duration ranged from 1 to 24 months, with a median duration of 6 months. Soy isoflavone supplementation doses ranged from 40 to 300 mg/day. Most studies did not report the dietary intake levels of soy isoflavone, while six studies provided a baseline dietary intake range of 1 to 31.7 mg/day of soy isoflavone [21, 26, 45, 51, 54, 55]. Nineteen studies employed parallel designs, while five studies used crossover designs.

### Quality assessment

The risk of bias in the included studies was evaluated using the Cochrane Collaboration’s tool, and the results can be found in Table S3. The studies were categorized as having low risk, unclear risk, or high risk based on seven criteria for bias assessment. In general, all studies demonstrated a low risk of bias for random sequence generation, and thirteen studies exhibited a low risk of bias for allocation concealment. All trials were deemed to have a low risk of bias concerning blinding of participants and personnel, as well as selective reporting. However, two studies were found to have a high risk of bias for blinding of outcome assessment. One article was identified as having a high risk of bias for incomplete data outcomes, while most studies displayed a low risk of bias for other potential sources of bias. The quality of evidence for SBP and DBP was evaluated as moderate quality, and further details of the GRADE framework for quality assessment can be found in Table 2 of Supplementary Material 3.

### Effect of soy isoflavone supplementation on SBP

**Fig. 2:**
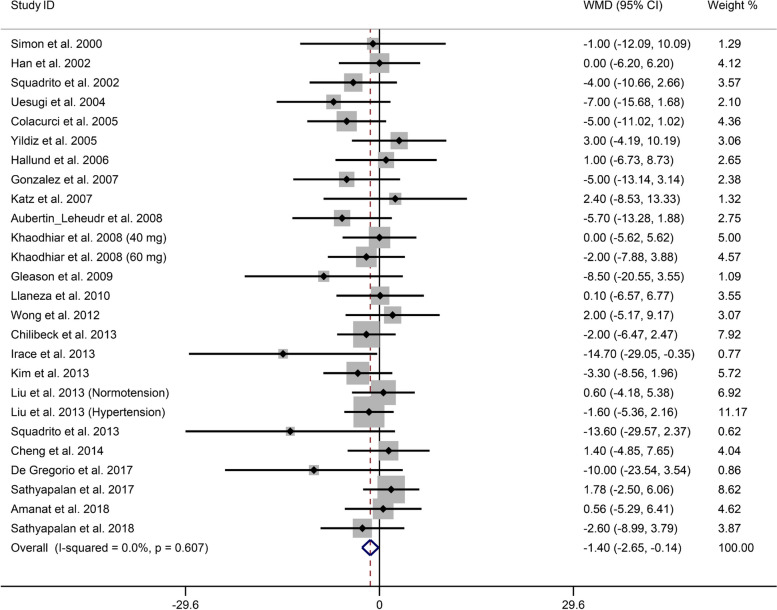
Forest plot for the effect of soy isoflavone supplementation on SBP. The pooled effect estimate was represented by the diamond. Data were presented as WMD and 95% CI using the fixed-effects model. Abbreviation: SBP, systolic blood pressure; WMD, weight mean differences; 95% CI, 95% confidence interval.

The overall results indicated that soy isoflavone supplementation significantly decreased SBP (WMD, -1.40 mmHg; 95% CI, -2.62 to -0.14 mmHg) (Fig. 2). The heterogeneity test revealed that SBP had low heterogeneity in the studies (*I*^2^ = 0.00%, 95% CI, 0 to 38.5%, *P* = 0.61), and a fixed-effects model was used to evaluate the pooled effects.

According to our subgroup analyses, the SBP was significantly lower in the group treated with soy isoflavone for at least 6 months and in the mixed-type soy isoflavone group (Table 3 of Supplementary Material 3). Furthermore, the group receiving soy isoflavone supplementation exhibited a significant reduction in SBP in studies involving healthy individuals and patients with metabolic syndrome, and in studies conducted with participants with prehypertension (Table 3 of Supplementary Material 3).

### Effect of soy isoflavone supplementation on DBP

**Fig. 3:**
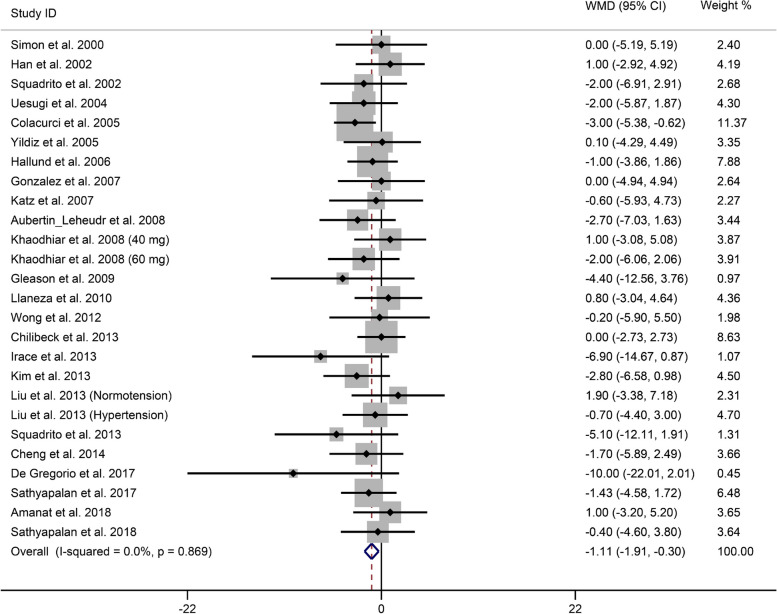
Forest plot for the effect of soy isoflavone supplementation on DBP. The pooled effect estimate was represented by the diamond. Data were presented as WMD and 95% CI using the fixed-effects model. Abbreviation: DBP, diastolic blood pressure; WMD, weight mean differences; 95% CI, 95% confidence interval.

The overall effect sizes showed that soy isoflavone supplementation significantly reduced in DBP (WMD, -1.11 mmHg; 95% CI, -1.91 to -0.30 mmHg) (Fig. 3). The heterogeneity test of combined studies revealed that DBP had low heterogeneity (*I*^2^ = 0.00%, 95% CI, 0 to 38.5%, *P* = 0.87), and a fixed effects model was used for meta-analysis.

According to our subgroup analyses, compared with those in the control group, the DBP in the soy isoflavone supplementation group significantly decreased after intervention for at least 6 months or in the mixed-type soy isoflavone group (Table 3 of Supplementary Material 3). Furthermore, DBP resulting from soy isoflavone supplementation was significantly lower in studies performed with participants with prehypertension. Similar results were found in healthy individuals and patients with metabolic syndrome (Table 3 of Supplementary Material 3).

### Meta-regression analyses

Meta-regression was conducted to investigate the linear relationship between dosage of soy isoflavone, duration of intervention, and alterations in blood pressure. A significant association between the dosage of soy isoflavone and its impact on SBP (*P*_linearity_ = 0.51) and DBP (*P*_linearity_ = 0.83) through supplementation was not identified. Moreover, the influence of soy isoflavone supplementation on SBP (*P*_linearity_ = 0.65) and DBP (*P*_linearity_ = 0.55) did not show a correlation with the duration of intervention (Table S4).

### The dose-response effect of soy isoflavone supplementation on blood pressure

**Fig. 4:**
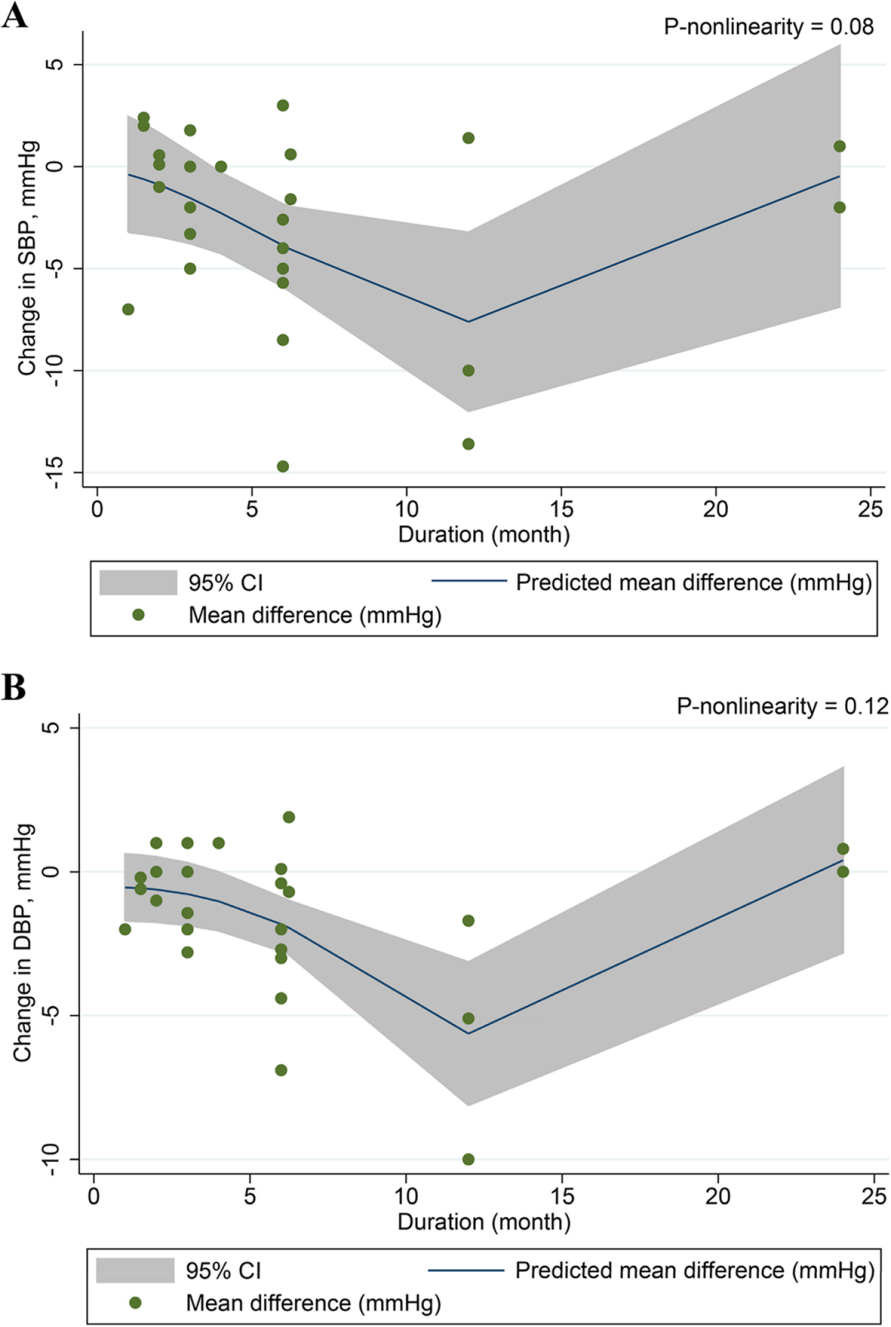
Non-linear dose-response relationship between intervention duration and unstandardized mean difference in SBP (A) and DBP (B). The 95% CI was presented in the shaded regions. Abbreviation: DBP, diastolic blood pressure; SBP, systolic blood pressure; 95% CI, 95% confidence interval.

**Fig. 5:**
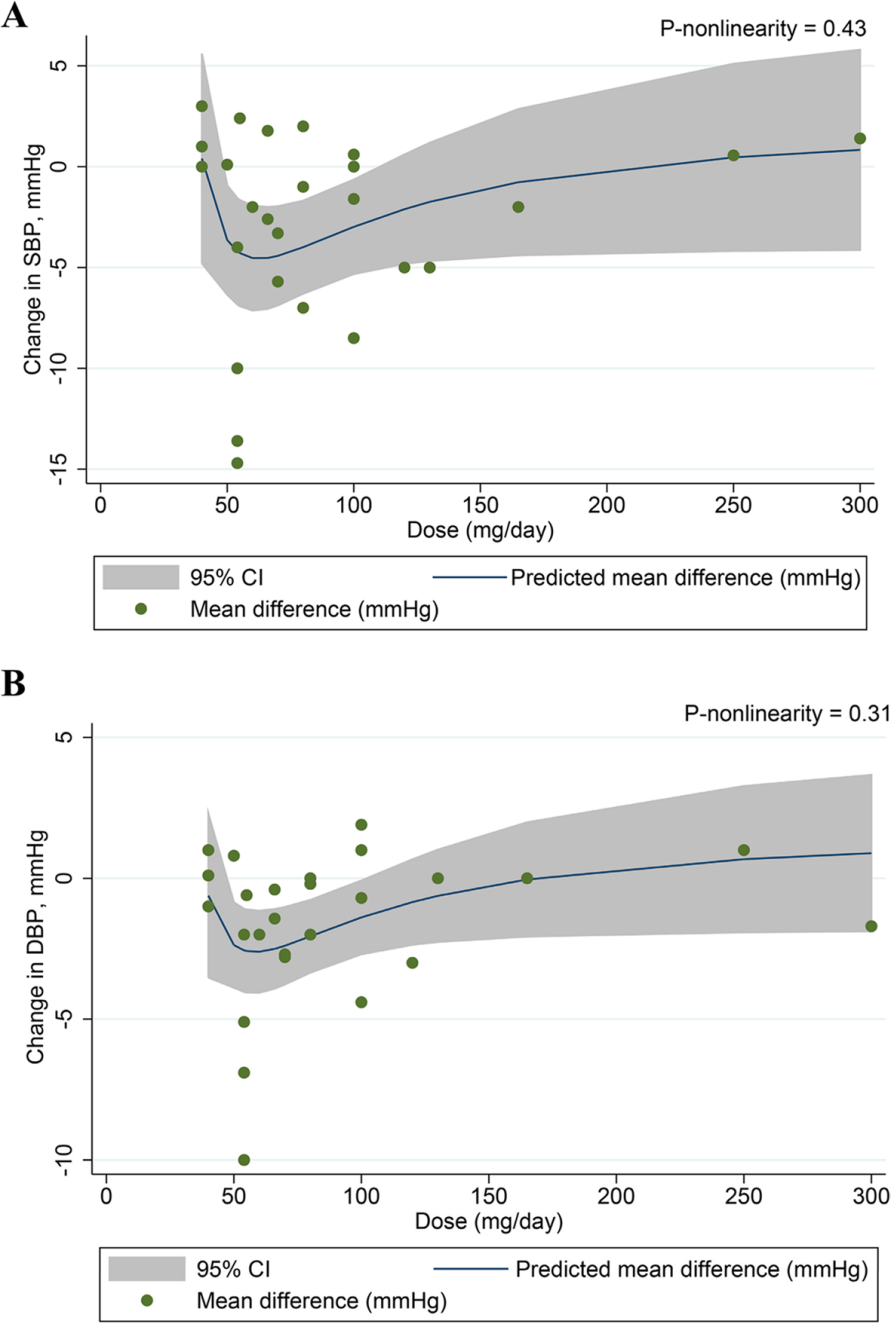
Non-linear dose-response relationship between dosage of soy isoflavone and unstandardized mean difference in SBP (A) and DBP (B). The 95% CI was presented in the shaded regions. Abbreviation: DBP, diastolic blood pressure; SBP, systolic blood pressure; 95% CI, 95% confidence interval.

After conducting a dose-response assessment, we found no significant non-linear association between intervention duration and SBP (*P*_non-linearity_ = 0.08) (Fig. 4A) or between intervention duration and DBP (*P*_non-linearity_ = 0.12) (Fig. 4B). We also failed to find a significant non-linear relationship between soy isoflavone dosage and SBP (*P*_non-linearity_ = 0.43) (Fig. 5A) or DBP (*P*_non-linearity_ = 0.31) (Fig. 5B).

### Sensitivity analyses

The results of sensitivity analyses showed that there was no significant influence on the overall pooled effect size of SBP or DBP by sequentially excluding one study (Figs. S1A and S1B).

### Publication bias

The assessment of publication bias indicated that the funnel plots for SBP and DBP were symmetrical, suggesting the absence of publication bias in the meta-analysis of the effect of soy isoflavone on SBP and DBP (Figs. S2A and S2B). Furthermore, the quantitative results of Egger’s linear regression test also supported this finding, as they did not reveal significant evidence of publication bias (SBP: *P* = 0.13; DBP: *P* = 0.33).

## Discussion

This meta-analysis examined the impact of soy isoflavone supplementation on blood pressure in adults, incorporating the latest studies. Our findings indicate that soy isoflavone supplementation led to a significant reduction in both SBP and DBP. Subgroup analyses further revealed that soy isoflavone supplementation resulted in decreased SBP and DBP among individuals undergoing long-term interventions (at least 6 months) and those receiving mixed-type soy isoflavone. Additionally, a significant reduction in both SBP and DBP was observed in both healthy participants and individuals with metabolic syndrome or prehypertension following soy isoflavone intake.

Current evidence has not determined whether soy isoflavone or soy protein combined with soy isoflavone can reduce blood pressure. A prior meta-analysis supported our findings [28]. It is worth noting that all the studies included in this meta-analysis were focused on assessing the impact of soy protein containing isoflavone on blood pressure. In order to clarify the direct influence of soy isoflavone on blood pressure regulation, we specifically analyzed studies with interventions that solely involved soy isoflavone in this meta-analysis. Furthermore, our results contradicted another meta-analysis that reported soy isoflavone significantly reduced SBP but had no effect on DBP [27]. This discrepancy in outcomes could be attributed to the inclusion of a larger number of studies and larger sample sizes, which enhanced the statistical power in our meta-analysis. Over the past decade, there have been numerous additions to the literature; hence, we made an effort to investigate the dose-response relationship between soy isoflavone supplementation and its effect on blood pressure. However, in this study, we did not observe a linear or non-linear dose-response relationship between the dosage of soy isoflavone supplementation or intervention duration and SBP and DBP. It is worth noting that most of the studies included various types of individual soy isoflavones, and their content was often unclear. Additionally, the metabolism and absorption of soy isoflavone can be influenced by factors such as gut microbiota, diet, and endogenous estrogen levels [10].

Factors such as race/ethnicity and isoflavone metabolites should also be considered when interpreting our results. After prolonged consumption of soy isoflavone, there was a more significant increase in the maximum concentration in plasma and the area under the plasma concentration-time curve values for daidzein and genistein in Caucasians compared to Asians. This suggests the existence of racial disparities in the pharmacokinetics and bioavailability of soy isoflavone [60]. Furthermore, the production of Equol, a metabolite of daidzein, is seen in only 25-30% of adults in Western countries, whereas it is produced by 60% of adults in Asian countries [61]. Research has indicated that equol possesses superior bioavailability and antioxidant activity compared to other soy isoflavones and may be responsible for the cardiovascular benefits associated with soy isoflavone. Therefore, it is imperative that future high-quality studies specifically focus on distinct racial groups and isoflavone metabolites to elucidate the potential influence of these factors on the antihypertensive effects of soy isoflavone.

Controlling blood pressure is a crucial approach to mitigating the risk of cardiovascular disease. Even a modest decrease of 5 mmHg in SBP can lead to a 10% reduction in the risk of major cardiovascular events and a 5% decrease in cardiovascular mortality [62]. Similarly, a 2 mmHg reduction in population DBP is associated with a 17% reduction in the prevalence of hypertension and a 6% lower risk of coronary heart disease [63]. Furthermore, even individuals with normal blood pressure benefit from a slight reduction in blood pressure, which diminishes the risk of cardiovascular disease [64]. It is worth noting that implementing antihypertensive strategies at the population level tends to be more cost-effective than individual strategies due to the high global incidence of hypertension [65]. Although the relatively small reductions in SBP and DBP observed in our study might not be significant clinically important, moderate soy isoflavone supplementation is a safe dietary intervention for the prevention of CVDs. Therefore, soy isoflavone supplementation may be regarded as a beneficial strategy for controlling blood pressure and reducing cardiovascular risk in adults in general, including prehypertensive individuals.

Subgroup analyses revealed that soy isoflavone supplementation significantly decreased blood pressure in participants who underwent an intervention for a duration of ≥6 months. This phenomenon was part presumably due to the time required to reduce blood pressure. Moreover, subgroup analyses determined that soy isoflavone supplementation was more efficacious in lowering blood pressure among participants with metabolic syndrome compared to other groups, including healthy individuals. Our subgroup analyses also revealed that supplementation with mixed types of soy isoflavones had a blood pressure-reducing effect. Previous research has speculated that different isoflavones may interact with each other in a synergistic or antagonistic manner [66]. For example, the synergistic effect of daidzein and genistein has been shown to improve male reproductive function [67]. However, the interaction among individual isoflavones in the context of cardiovascular health remains unclear and warrants further investigation.

The mechanism underlying the blood pressure-lowering effect of soy isoflavones has been studied and reported. Soy isoflavones have the potential to promote vasodilation by influencing the endothelium and participating in the maintenance of vascular homeostasis. Genistein and daidzein, in particular, have been shown to enhance the secretion of nitric oxide in endothelial cells, leading to vasodilation, reduced vascular resistance, and ultimately, a decrease in blood pressure [19, 68, 69]. Additionally, soy isoflavones have been found to combat hypertension by influencing components within the renin-angiotensin-aldosterone system. Treatment with daidzein and genistein significantly decreased the activity and expression of ACE and had significant hypotensive effects [20, 70, 71]. Furthermore, genistein supplementation was found to inhibit carotid baroreceptor activity, with the proposed mechanism being the suppression of protein tyrosine kinase activity and reduction of Ca^2+^ influx through stretch-activated channels [72]. Baroreceptors are widely recognized for their critical role in the long-term regulation of blood pressure [73]. The aforementioned evidence suggests that genistein influences the regulation of vasodilation via peripheral or carotid sinus baroreceptors, potentially serving as another mechanism for reducing blood pressure.

The present meta-analysis has several strengths. First, we conducted a comprehensive quantitative review on the effect of soy isoflavone supplementation alone on blood pressure. To our knowledge, previous evidence, including that from multiple RCTs and two meta-analyses, has shown conflicting findings. Second, based on various factors, our study conducted subgroup analyses to explore the differences between different subgroups. However, several limitations should be considered in the present study. Several factors need to be considered when interpreting our results. Firstly, the inclusion of studies involving participants with varying health characteristics, such as diabetes, metabolic syndrome, and non-alcoholic fatty liver, may have introduced some confounding into our findings. However, it is worth noting that our overall results were largely consistent with those of the majority population subgroup. Secondly, the majority of the included RCTs in this study employed different types and proportions of soy isoflavones, making it challenging to determine which specific species or optimal proportion is most beneficial for blood pressure regulation. Thirdly, the clinical effects of soy isoflavones can be influenced by the individual's ability to convert soy isoflavone into more potent metabolites like equol. The composition of gut microbiota varies among individuals, leading to a high degree of variability in equol production. Fourthly, blood pressure, considered a secondary outcome in some of the included studies, may have an impact on the reliability of the measurements. Lastly, it is important to note that the majority of the studies were conducted among women and middle-aged and older adults (≥40 years old). To gain a more comprehensive understanding of the effect of soy isoflavones on blood pressure, further research involving men or young adults is still warranted.

## Conclusions

To sum up, this meta-analysis has demonstrated that soy isoflavone supplementation, when administered alone, has a significant and favorable effect on reducing both SBP and DBP. This effect was especially pronounced in trials with intervention durations of at least 6 months, among participants receiving mixed types of soy isoflavones, as well as in healthy individuals and those with metabolic syndrome. Additionally, soy isoflavone supplementation showed a noteworthy antihypertensive effect in adults with prehypertension. These findings suggest that soy isoflavone supplementation may be a potential strategy for hypertension prevention. However, more epidemiological studies involving diverse populations with varying health conditions are necessary to provide further corroborative evidence. Additionally, conducting more high-quality RCTs is essential to determine the optimal quantity and proportion of soy isoflavones for maximum benefit.

### Supplementary Information

**Supplementary Material 1.**

**Supplementary Material 2.**

**Supplementary Material 3.**

## Data Availability

The datasets analyzed during the current study are presented in the manuscript.

## Abbreviations

ACE: Angiotensin-converting enzyme
BMI: Body mass index
CVDs: Cardiovascular diseases
CI: Confidence interval
DBP: Diastolic blood pressure
GRADE: Grading of Recommendations Assessment, Development and Evaluation
RCTs: Randomized controlled trials
SBP: Systolic blood pressure
SEM: Standard error of mean
SD: Standard deviations
WMD: Weighted mean difference
