# Correction: Effect of soy isoflavone supplementation on blood pressure: a meta-analysis of randomized controlled trials

**DOI:** 10.1186/s12937-024-00941-5

**Published:** 2024-05-17

**Authors:** Lifu Lei, Suocheng Hui, Yushi Chen, Hongjia Yan, Jian Yang, Shiwen Tong

**Affiliations:** 1 Department of Clinical Nutrition, The Second Affiliated Hospital of Chongqing Medical University, Chongqing, 400016 China; 2 Department of Clinical Nutrition, The People’s Hospital of Chongqing Liang Jiang New Area, Chongqing, 401135 China; 3 Department of Clinical Nutrition, The Third Affiliated Hospital of Chongqing Medical University, Chongqing, 410020 China; 4 Research Center for Metabolic and Cardiovascular Diseases, The Third Affiliated Hospital of Chongqing Medical University, Chongqing, 410020 China

**Correction: Nutr J 23, 32 (2024)**

**https://doi.org/10.1186/s12937-024-00932-6**

Following publication of the original article [1], the author reported that Tables 1, 2, 3 were not captured.

The tables are given below and captured as Supplementary files in the original article.

The original article has been updated.

**Table 1:** Characteristics of twenty-four randomized controlled trials included in the meta-analysis

First author, year	Country	Duration of intervention (months)	Study design	Sample size (isoflavone/control)	Mean age (year)	BMI (kg/m^2^)	Women (%)	Population		Intervention	
										**Soy isoflavone group**	**Control group**
Simon et al. 2000 [42]	Australia	2	Crossover	20/20	59.0	26.8	100.0	Healthy postmenopausal women		80 mg soy isoflavone (PhytoLife 1)	Placebo (NR)
Han et al. 2002 [43]	Brazil	4	Parallel	40/40	48.5	24.9	100.0	Postmenopausal women		100 mg soy isoflavone (50.3 mg soy protein and 33.3 mg isoflavone per capsule)	Placebo (50.3 mg soy protein and 33.3 mg glucose per capsule)
Squadrito et al. 2002 [44]	Italy	6	Parallel	30/30	56.0	NR	100.0	Healthy Postmenopausal women		54 mg soy isoflavone (genistein)	Placebo (NR)
Uesugi et al. 2004 [45]	Japan	1	Crossover	58/58	58.0	23.0	100.0	Climacteric women		40 mg soy isoflavone (3.4 mg daidzein, 0.9 mg genistein, and 2.7 mg glycitein per gram of tablet)	Placebo (same matrix without isoflavone)
Colacurci et al. 2005 [24]	Italy	6	Parallel	29/28	55.2	25.9	100.0	Healthy postmenopausal women		120 mg soy isoflavone (60 mg genistein and 60 mg daidzein)	Placebo (NR)
Yildiz et al. 2005 [25]	Turkey	6	Parallel	20/20	50.0	27.2	100.0	Healthy postmenopausal women		40 mg soy isoflavone (genistein)	Placebo (NR)
Hallund et al. 2006 [46]	Denmark	2	Crossover	30/28	57.0	24.0	100.0	Healthy postmenopausal women		Cereal bars with 50 mg soy isoflavone (genistein:daidzein ratio of 2:1)	Placebo (cereal bars)
Gonzalez et al. 2007 [47]	United Kingdom	3	Crossover	26/26	NR	31.0	100.0	Postmenopausal women with type 2 diabetes		132 mg soy isoflavone (53% genistein, 37% daidzein, and 10% glycitein)	Placebo (microcrystalline cellulose)
Katz et al. 2007 [48]	United States	1.5	Crossover	22/22	58.5	27.6	100.0	Healthy postmenopausal women		55 mg soy isoflavone (daidzein and genistein)	Placebo (NR)
Aubertin-Leheudr et al. 2008 [49]	Canada	6	Parallel	25/25	57.4	32.0	100.0	Postmenopausal obese women		70 mg soy isoflavone (44 mg daidzein, 16 mg glycitein, and 10 mg genistein)	Placebo (NR)
Khaodhiar et al. 2008 [50]	United States	3	Parallel	97/45	53.1	28.6	100.0	Healthy menopausal women		40 or 60 mg soy isoflavone (70% daidzein, 10% genistein, and 20% glycitein)	Placebo (NR)
Gleason et al. 2009 [51]	United States	6	Parallel	15/15	73.7	NR	50.0	Healthy men and postmenopausal women		100 mg soy isoflavone (85% daidzein and genistein)	Placebo (maltodextrin and caramel food colour)
Llaneza et al. 2010 [52]	Spain	24	Parallel	58/58	56.4	30.0	100.0	Postmenopausal women with insulin resistance		Mediterranean diet with 40 mg soy isoflavone (NR)	Placebo (mediterranean diet)
Wong et al. 2012 [53]	United States	1.5	Parallel	12/12	55.7	25.4	100.0	Postmenopausal women		80 mg soy isoflavone (22.0 mg daidzein, 13.5 mg glycitein, and 5.0 mg genistein per tablet)	Placebo (1.0 mg aglycone equivalent per tablet)
Chilibeck et al. 2013 [54]	Canada	24	Parallel	90/88	56.6	27.1	100.0	Postmenopausal women		165 mg soy isoflavone (105 mg aglycone equivalent)	Placebo (dicalcium phosphate, magnesium stearate, and sorbitol)
Irace et al. 2013 [26]	Italy	6	Parallel	10/10	58.8	31.8	100.0	Postmenopausal women with metabolic syndrome		54 mg soy isoflavone (genistein)	Placebo (NR)
Kim et al. 2013 [55]	Korea	3	Parallel	42/43	53.6	23.2	100.0	Postmenopausal women with type 2 diabetes		70 mg soy isoflavone (38.0 mg glycitein, 20 mg daidzein, and 12.4 mg genistein)	Placebo (NR)
Liu et al. 2013 [21]	China	6.25	Parallel	60/60	NR	NR	100.0	Postmenopausal women		15 g milk protein and 100 mg isoflavone (35 mg daidzein, 59 mg genistein, and 4 mg glycitein)	Placebo (15 g milk protein)
Squadrito et al. 2013 [22]	Italy	12	Parallel	60/60	55.5	31.8	100.0	Postmenopausal women with metabolic syndrome		54 mg soy isoflavone (genistein)	Placebo (NR)
Cheng et al. 2015 [56]	China	12	Parallel	41/41	56.6	23.0	100.0	Postmenopausal women		300 mg isoflavone aglycone (NR)	Placebo (NR)
De Gregorio et al. 2017 [57]	Italy	12	Parallel	11/11	NR	30.3	100.0	Postmenopausal women with metabolic syndrome		54 mg soy isoflavone (genistein)	Placebo (NR)
Sathyapalan et al. 2017 [58]	United Kingdom	3	Parallel	100/100	52.0	31.7	0.0	Men with type 2 diabetes		15 g soy protein with 66 mg isoflavone (54% genistein, 35% daidzein, and 11% glycitein)	Placebo (15 g soy protein)
Amanat et al. 2018 [59]	Iran	2	Parallel	41/41	43.6	28.5	24.4	Patients with non-alcoholic fatty liver disease	250 mg soy isoflavone (genistein)		Placebo (cornstarch)
Sathyapalan et al. 2018 [23]	United Kingdom	6	Parallel	60/60	52.0	25.5	100.0	Early menopause women	15 g soy protein with 66 mg isoflavone (54% genistein, 35% daidzein, and 11% glycitein)		Placebo (15 g soy protein)

*Abbreviation*:* BMI* Body mass index, *NR* not report

**Table 2:** Assessment of quality of evidence for outcomes using the GRADE

	**Quality assessment**							**No. of patients**			**Quality of evidence** **(GRADE)**
**Outcomes**	**No of studies**	**Study design**	**Risk of bias**	**Imprecision**	**Inconsistency**	**Indirectness**	**Publication bias**	**Intervention**	**Control**	**WMD (95%CI)**	
SBP	26	Randomized trials	No serious risk of bias ^a^	No serious imprecision ^b^	No serious inconsistency ^c^	Serious indirectness ^d^	No serious limitation ^e^	1005	940	-1.40 (-2.65, -0.14)	⨁⨁⨁◯ Moderate
DBP	26	Randomized trials	No serious risk of bias	No serious imprecision	No serious inconsistency	Serious indirectness	No serious limitation	1005	940	-1.11 (-1.91, -0.30)	⨁⨁⨁◯ Moderate

The quality of evidence was evaluated at 4 levels using GRADE (high, moderate, low, very low). ^a^ No downgrade for risk of bias, as most studies were assessed as low risk of bias. ^b^ No downgrade for imprecision, as the optimal information size was met and the 95%CI did not include the null value. ^c^ No downgrade for inconsistency, as there was a low of heterogeneity (SBP: *I* ^2^ = 0.0%, *P* = 0.61; DBP: *I* ^2^ = 0.0%, *P* = 0.87). ^d^ Downgraded for indirectness, as the intake of sodium and potassium was not provided, which may have confounding effects. ^e^ No downgrade for publication bias, as there was no publication bias evaluated by the funnel plots and Egger's test. Abbreviation: *DBP* diastolic blood pressure, *SBP* systolic blood pressure, *GRADE* Grading of Recommendations Assessment, Development and Evaluation, *WMD* weighted mean difference, *95% CI* 95% confidence interval.

**Table 3:** Subgroup analyses of soy isoflavone supplementation on blood pressure in adults

**Subgroup**		**SBP**					**DBP**				
			**Test of heterogeneity**					**Test of heterogeneity**			
	**No. of studies**	**Net change** **(95% CI)**	***P*** ^**a**^	***I*** ^**2**^ **(%)**	***P*** ^**b**^	**No. of studies**	**Net change** **(95% CI)**	***P*** ^**a**^		***I*** ^**2**^ ** (%)**	***P*** ^**b**^
**Resting blood pressure status**											
Normotension	6	-0.77 (-3.21, 1.68)	0.54	0.00	0.73	6	-0.95 (-2.55, 1.77)	0.24		0.00	0.68
Prehypertension	16	-1.99 (-3.64, -0.35)	0.02	0.00	0.60	16	-1.05 (-2.08, -0.02)	0.04		0.00	0.59
Hypertension	2	-1.65 (-7.18, 3.89)	0.56	59.00	0.12	2	-1.43 (-4.63, 1.77)	0.38		0.00	0.61
**Dose of soy isoflavone (mg/day)**											
< 50	3	1.11 (-2.74, 4.95)	0.57	0.00	0.81	3	-0.24 (-2.31, 1.82)	0.81		0.00	0.72
≥ 50, ≤ 100	18	-1.67 (-3.22, -0.12)	0.03	9.10	0.46	18	-1.28 (-2.36, -0.19)	0.02		0.00	0.82
> 100	5	-1.77 (-4.37, 0.83)	0.18	0.00	0.51	5	-1.23 (-2.69, 0.24)	0.10		6.40	0.37
**Duration of follow-up** **(months)**											
< 6	12	-0.61 (-2.46, 1.24)	0.52	0.00	0.82	12	-0.75 (-1.91, 0.40)	0.20		0.00	0.95
≥ 6	14	-2.08 (-3.80, -0.36)	0.02	10.30	0.34	14	-1.43 (-2.54, -0.32)	0.01		0.00	0.51
**Types of soy isoflavone**											
Genistein	6	-2.29 (-5.71, 1.14)	0.19	46.20	0.10	6	-1.59 (-3.86, 0.68)	0.17		25.30	0.24
Mixed types of soy isoflavone	19	-1.39 (-2.78, -0.01)	0.04	0.00	0.84	19	-1.00 (-1.89, -0.13)	0.02		0.00	0.92
**Health status**											
Health	16	-1.68 (-3.33, -0.04)	0.04	0.00	0.63	16	-1.07 (-2.04, -0.10)	0.03		0.00	0.88
Diabetes	3	-0.92 (-4.00, 2.15)	0.57	39.00	0.19	3	-1.61 (-3.78, 0.57)	0.15		0.00	0.67
Metabolic syndrome	3	-12.60 (-20.98, -4.21)	< 0.01	0.00	0.89	3	-6.55 (-11.33, -1.78)	0.01		0.00	0.78
Hypertension	2	-1.65 (-7.18, 3.89)	0.56	59.00	0.12	2	-1.43 (-4.63, 1.77)	0.38		0.00	0.61
Non-alcoholic fatty liver disease	1	0.56 (-5.29, 6.41)	0.85			1	1.00 (-3.20, 5.20)	0.64			
Insulin resistance	1	0.10 (-6.57, 6.77)	0.98			1	0.80 (-3.05, 4.65)	0.68			
**Baseline BMI** **(kg/m** ^**2**^ **)**											
< 25	5	-1.04 (-3.67, 1.59)	0.44	0.00	0.45	5	-0.99 (-2.84, 0.85)	0.29	0.00		0.49
≥ 25	18	-1.35 (-2.86, 0.17)	0.08	0.00	0.48	18	-0.82 (-1.72, 0.08)	0.07	0.00		0.66
**Mean age**											
< 50	2	0.30 (-3.96, 4.55)	0.89	0.00	0.90	2	1.00 (-1.87, 3.87)	0.49	0.00		1.00
≥ 50	23	-1.30 (-2.61, 0.01)	0.05	1.90	0.45	23	-1.28 (-2.13, -0.43)	0.01	0.00		0.95
**Gender**											
Female	23	-1.73 (-3.09, -0.37)	0.01	0.00	0.68	23	-1.26 (-2.17, -0.34)	0.01	0.00		0.80
Male	1	1.78 (-2.50, 3.66)	0.42			1	-1.43 (-4.58, 1.72)	0.37			
Both sexes	2	-1.17 (-6.43, 4.10)	0.66	43.00	0.16	2	-0.13 (-3.87, 3.60)	0.95	24.80		0.25

Abbreviation: *BMI* Body mass index, *DBP* diastolic blood pressure, *SBP* systolic blood pressure, *95 CI* 95 confidence interval.

^a^ *P* values for difference between subgroups. ^b^ *P* values for heterogeneity test within subgroups.
