# Supplementary material for: Effect of soy isoflavone supplementation on blood pressure: a meta-analysis of randomized controlled trials
Source: Nutr J. 2024 Mar 7;23:32. doi: 10.1186/s12937-024-00932-6 (PMC10918941; doi:10.1186/s12937-024-00932-6)
Supplement: Supplementary file 1 — Supplementary Material 1. [file 12937_2024_932_MOESM1_ESM.doc]

Effect of soy isoflavone supplementation on blood pressure: a meta-analysis of randomized controlled trials

Lifu Lei^1#^, Suocheng Hui^3#^, Yushi Chen^1^, Hongjia Yan^3^, Jian Yang^2,4*^, [Shiwen Tong](https://pubmed.ncbi.nlm.nih.gov/?sort=date&term=Tong+S&cauthor_id=35917968)^1*^

^1^Department of Clinical Nutrition, The Second Affiliated Hospital of Chongqing Medical University, Chongqing, China, 400016

^2^Department of Clinical Nutrition, The Third Affiliated Hospital of Chongqing Medical University, Chongqing, China, 410020

^3^Department of Clinical Nutrition, The People’s Hospital of Chongqing Liang Jiang New Area, Chongqing, China, 401135

^4^Research Center for Metabolic and Cardiovascular Diseases, The Third Affiliated Hospital of Chongqing Medical University, Chongqing, China, 410020

#These authors contributed equally to this work.

***Corresponding authors:**

Dr. [Shiwen Tong](https://pubmed.ncbi.nlm.nih.gov/?sort=date&term=Tong+S&cauthor_id=35917968), Email: tswcqmu@cqmu.edu.cn

Dr. Jian Yang, Email: jianyang@hospital.cqmu.edu.cn

Table S1 Full search strategy for Pubmed

| **Search strategy** | |
| --- | --- |
| Pubmed |  |
| #1 | ((((((Soy) OR (Soybean[MeSH Terms])) OR (Soy Beans[Title/Abstract])) OR (Bean, Soy[Title/Abstract])) OR (Beans, Soy[Title/Abstract])) OR (Soy Bean[Title/Abstract])) OR (Glycine max[Title/Abstract]) |
| #2 | (((((((((((((((Isoflavone[MeSH Terms]) OR (Isoflavone Derivatives[Title/Abstract])) OR (Derivatives, Isoflavone[Title/Abstract])) OR (Isoflavone Derivative[Title/Abstract])) OR (Derivative, Isoflavone[Title/Abstract])) OR (Isoflavone[Title/Abstract])) OR (Homoisoflavones[Title/Abstract])) OR (3-Benzylidene-4-Chromanone[Title/Abstract])) OR (3 Benzylidene 4 Chromanone[Title/Abstract])) OR (Homoisoflavone[Title/Abstract])) OR (3-Benzylidene-4-Chromanones[Title/Abstract])) OR (3 Benzylidene 4 Chromanones[Title/Abstract])) OR (3-Benzylchroman-4-Ones[Title/Abstract])) OR (3 Benzylchroman 4 Ones[Title/Abstract])) OR (3-Benzylchroman-4-One[Title/Abstract])) OR (3 Benzylchroman 4 One[Title/Abstract]) |
| #3 | (((((((((((Phytoestrogen[MeSH Terms]) OR (Plant Estrogen[Title/Abstract])) OR (Estrogen, Plant[Title/Abstract])) OR (Phytoestrogen[Title/Abstract])) OR (Plant Estrogens[Title/Abstract])) OR (Estrogens, Plant[Title/Abstract])) OR (Phyto-Estrogen[Title/Abstract])) OR (Phyto Estrogen[Title/Abstract])) OR (Estrogen-Like Plant Extracts[Title/Abstract])) OR (Estrogen Like Plant Extracts[Title/Abstract])) OR (Extracts, Estrogen-Like Plant[Title/Abstract])) OR (Plant Extracts, Estrogen-Like[Title/Abstract]) |
| #4 | (Genistein[MeSH Terms]) OR (Genestein[Title/Abstract]) |
| #5 | (Daidzein) OR (Diadzein[Title/Abstract]) |
| #6 | (Glycitein) |
| #7 | ((((((((((((((((Soy protein[MeSH Terms]) OR (Soy Bean Proteins[Title/Abstract])) OR (Soybean Protein[Title/Abstract])) OR (Protein, Soybean[Title/Abstract])) OR (Soy Bean Protein[Title/Abstract])) OR (Bean Protein, Soy[Title/Abstract])) OR (Protein, Soy Bean[Title/Abstract])) OR (Dietary Soybean Proteins[Title/Abstract])) OR (Dietary Soybean Protein[Title/Abstract])) OR (Protein, Dietary Soybean[Title/Abstract])) OR (Proteins, Dietary Soybean[Title/Abstract])) OR (Soybean Protein, Dietary[Title/Abstract])) OR (Soybean Proteins, Dietary[Title/Abstract])) OR (Soy Proteins[Title/Abstract])) OR (Protein, Soy[Title/Abstract])) OR (Proteins, Soy[Title/Abstract])) OR (Soy Protein[Title/Abstract]) |
| #8 | #1 OR #2 OR #3 OR #4 OR #5 OR #6 OR #7 |
| #9 | ((((((((Blood pressure[MeSH Terms]) OR (Pressure, Blood[Title/Abstract])) OR (Diastolic Pressure[Title/Abstract])) OR (Pressure, Diastolic[Title/Abstract])) OR (Pulse Pressure[Title/Abstract])) OR (Pressure, Pulse[Title/Abstract])) OR (Systolic Pressure[Title/Abstract])) OR (Pressure, Systolic[Title/Abstract])) OR (Pressures, Systolic[Title/Abstract]) |
| #10 | ((((Hypertension[MeSH Terms]) OR (Blood Pressure, High[Title/Abstract])) OR (Blood Pressures, High[Title/Abstract])) OR (High Blood Pressure[Title/Abstract])) OR (High Blood Pressures[Title/Abstract]) |
| #11 | (Hypertensive) |
| #12 | ((((Hypotension[MeSH Terms]) OR (Vascular Hypotension[Title/Abstract])) OR (Low Blood Pressure[Title/Abstract])) OR (Blood Pressure, Low[Title/Abstract])) OR (Hypotension, Vascular[Title/Abstract]) |
| #13 | (Hypotensive) |
| #14 | (((((((((((((((((((((((((Antihypertensive agents[MeSH Terms]) OR (Agents, Antihypertensive[Title/Abstract])) OR (Antihypertensive Agent[Title/Abstract])) OR (Agent, Antihypertensive[Title/Abstract])) OR (Anti-Hypertensive Agent[Title/Abstract])) OR (Agent, Anti-Hypertensive[Title/Abstract])) OR (Anti Hypertensive Agent[Title/Abstract])) OR (Anti-Hypertensive Drug[Title/Abstract])) OR (Anti Hypertensive Drug[Title/Abstract])) OR (Drug, Anti-Hypertensive[Title/Abstract])) OR (Antihypertensive Drug[Title/Abstract])) OR (Drug, Antihypertensive[Title/Abstract])) OR (Antihypertensives[Title/Abstract])) OR (Anti-Hypertensive Agents[Title/Abstract])) OR (Agents, Anti-Hypertensive[Title/Abstract])) OR (Anti Hypertensive Agents[Title/Abstract])) OR (Anti-Hypertensive Drugs[Title/Abstract])) OR (Anti Hypertensive Drugs[Title/Abstract])) OR (Drugs, Anti-Hypertensive[Title/Abstract])) OR (Anti-Hypertensives[Title/Abstract])) OR (Anti Hypertensives[Title/Abstract])) OR (Antihypertensive Drugs[Title/Abstract])) OR (Drugs, Antihypertensive[Title/Abstract])) OR (Anti-Hypertensive[Title/Abstract])) OR (Anti Hypertensive[Title/Abstract])) OR (Antihypertensive[Title/Abstract]) |
| #15 | #9 OR #10 OR #11 OR #12 OR #13 OR #14 |
| #16 | #8 AND #15 |

Table S2 Baseline and outcome of twenty-four randomized controlled trials included in the meta-analysisa

| **First author, year** | **SBP (mmHg)** | | **DBP (mmHg)** | |
| --- | --- | --- | --- | --- |
|  | **Soy isoflavone** | **Control** | **Soy isoflavone** | **Control** |
| Simon et al.  2000 [1] | Baseline (135.00 ± 17.89), outcome (-10.00 ± 17.89) | Baseline (1.30± 17.89), outcome (-9.00 ± 17.89) | Baseline (85.00 ± 8.94), outcome (-5.00 ± 7.75) | Baseline (85.00 ± 8.94), outcome (-5.00 ± 8.95) |
| Han et al.  2002 [2] | Baseline (131.00 ± 12.65), outcome (0.00 ± 10.95) | Baseline (133.00 ± 18.97), outcome (0.00 ± 16.73) | Baseline (84.00 ± 6.32), outcome (1.00 ± 6.32) | Baseline (84.00 ± 12.65), outcome (0.00 ± 10.95) |
| Squadrito et al. 2002 [3] | Baseline (113.00 ±11.00), outcome (-3.00 ± 12.12) | Baseline (112.00 ± 13.00), outcome (1.00 ± 14.11) | Baseline (80.00 ± 7.00), outcome (-1.00 ± 10.44) | Baseline (77.00 ± 10.00), outcome (1.00 ± 8.89) |
| Uesugi et al.  2004 [4] | Baseline (140.00 ± 24.00), outcome (-13.00 ± 21.93) | Baseline (140.00 ± 24.00), outcome (-6.00 ± 25.63) | Baseline (84.00± 11.00), outcome (-6.00 ± 9.64) | Baseline (84.00± 11.00), outcome (-4.00 ± 11.53) |
| Colacurci et al. 2005 [5] | Baseline (122.00 ± 10.00), outcome (-3.00 ± 10.53) | Baseline (119.00 ± 13.00), outcome (2.00 ± 12.53) | Baseline (79.00 ± 4.00), outcome (-1.00 ± 4.58) | Baseline (77.00 ± 5.00), outcome (2.00 ± 4.58) |
| Yildiz et al.  2005 [6] | Baseline (123.00 ±14.50), outcome (3.80 ± 12.59) | Baseline (130.00 ± 12.10), outcome (0.80 ± 10.51) | Baseline (77.00 ±6.60), outcome (1.10 ± 7.09) | Baseline (76.50 ± 7.30), outcome (1.00 ± 7.06) |
| Hallund et al.  2006 [7] | Baseline (123.00 ±16.43), outcome (-2.00 ± 17.23) | Baseline (124.00 ± 16.43), outcome (-3.00 ± 17.23) | Baseline (75.00 ±5.29), outcome (0.00 ± 5.55) | Baseline (76.00 ± 5.29), outcome (-1.00 ± 5.55) |
| Gonzalez et al. 2007 [8] | Baseline (130.00 ± 16.00), outcome (-1.00 ± 14.42) | Baseline (130.00± 15.00), outcome (4.00 ±15.52) | Baseline (73.00 ± 9.00), outcome (1.00 ± 9.00) | Baseline (75.00± 10.00), outcome (1.00 ± 9.17) |
| Katz et al.  2007 [9] | Baseline (126.90 ± 17.50), outcome (2.60 ± 18.18) | Baseline (126.90 ± 17.50), outcome (0.02 ± 18.82) | Baseline (71.50 ± 8.70), outcome (-1.60 ± 8.60) | Baseline (71.50 ± 8.70), outcome (-1.00 ± 9.42) |
| Aubertin-Leheudr et al. 2008 [10] | Baseline (125.10 ±15.00), outcome (-3.20 ± 15.80) | Baseline (126.40 ± 10.50), outcome (2.50 ± 11.15) | Baseline (79.00 ± 8.70), outcome (-2.30 ± 8.75) | Baseline (77.60 ± 6.30), outcome (0.40 ± 6.74) |
| Khaodhiar et al. 2008 (40 mg) [11] | Baseline (122.00 ± 14.00), outcome (-3.00 ± 13.53) | Baseline (120.00 ± 13.00), outcome (-3.00 ± 14.11) | Baseline (76.00 ± 10.00), outcome (-1.00 ± 10.54) | Baseline (76.00 ± 9.00), outcome (-2.00 ± 9.54) |
| Khaodhiar et al. 2008 (60 mg) [11] | Baseline (121.00 ± 15.00), outcome (-5.00 ± 15.00) | Baseline (120.00 ± 13.00), outcome (-3.00 ± 14.11) | Baseline (78.00 ± 10.00), outcome (-4.00 ± 10.54) | Baseline (76.00 ± 9.00), outcome (-2.00 ± 9.54) |
| Gleason et al.  2009 [12] | Baseline (NR ±NR), outcome (-5.80 ± 19.20) | Baseline (NR ± NR), outcome (2.70 ±14.10) | Baseline (NR ± NR), outcome (-5.00 ± 11.30) | Baseline (NR ± NR), outcome (-0.60 ± 11.50) |
| Llaneza et al.  2010 [13] | Baseline (131.00 ±14.35), outcome (-3.10 ± 17.09) | Baseline (128.00 ± 16.20), outcome (-3.20 ± 15.18) | Baseline (77.30 ±9.51), outcome (2.80 ± 8.98) | Baseline (75.90 ± 10.80), outcome (2.00 ± 9.60) |
| Wong et al.  2012 [14] | Baseline (140.10 ± 8.30), outcome (-2.80 ± 9.08) | Baseline (140.70± 7.40), outcome (-4.80 ± 8.85) | Baseline (82.80 ± 6.20), outcome (-2.90 ± 6.10) | Baseline (82.20± 8.70), outcome (-2.70 ± 8.02) |
| Chilibeck et al. 2013 [15] | Baseline (122.00 ± 14.00), outcome (-1.00 ± 14.32) | Baseline (122.00 ± 15.00), outcome (1.00 ± 16.50) | Baseline (79.00 ± 9.00), outcome (-1.00 ± 9.55) | Baseline (79.00 ± 10.00), outcome (-1.00 ± 9.44) |
| Irace et al.  2013 [16] | Baseline (133.60 ±18.10), outcome (-2.40 ± 19.53) | Baseline (121.40 ± 14.00), outcome (12.30 ± 12.42) | Baseline (81.50 ±9.10), outcome (-2.80 ± 8.12) | Baseline (79.30 ± 10.50), outcome (4.10 ± 9.56) |
| Kim et al.  2013 [17] | Baseline (116.10 ± 14.30), outcome (-5.30 ± 12.97) | Baseline (117.70± 12.10), outcome (-2.00 ± 11.72) | Baseline (74.60 ± 10.00), outcome (-3.00 ± 9.07) | Baseline (76.20± 9.50), outcome (-0.20 ± 8.71) |
| Liu et al. 2013  (Normotension)  [18] | Baseline (107.30 ± 7.60), outcome (1.00 ± 6.30) | Baseline (109.90 ±9.00), outcome (0.40 ± 7.60) | Baseline (69.00 ± 4.80), outcome (-0.20 ± 8.70) | Baseline (72.00 ± 8.00), outcome (-2.10 ± 6.70) |
| Liu et al. 2013  (Hypertension) [18] | Baseline (133.30 ± 10.10), outcome (-4.30 ± 9.10) | Baseline (131.60 ± 14.30), outcome (-2.70 ± 8.80) | Baseline (81.90 ± 8.70), outcome (-5.80 ± 8.30) | Baseline (72.00 ± 8.70), outcome (-5.10 ± 9.30) |
| Squadrito et al. 2013 [19] | Baseline (135.70 ± 31.94), outcome (-12.00 ± 14.60) | Baseline (135.00 ± 15.87), outcome (1.60 ± 16.40) | Baseline (78.70 ± 8.71), outcome (-4.20 ± 19.50) | Baseline (78.10 ± 13.36), outcome (0.90 ± 19.70) |
| Cheng et al.  2014 [20] | Baseline (119.00 ± 13.80), outcome (-1.90 ± 12.67) | Baseline (117.50± 16.60), outcome (-3.30 ± 16.03) | Baseline (77.30 ± 9.30), outcome (-3.60 ± 8.42) | Baseline (74.60± 11.10), outcome (-1.90 ± 10.81) |
| De Gregorio et al. 2017 [21] | Baseline (130.00 ± 38.00), outcome (-10.00 ± 17.50) | Baseline (130.00 ± 30.00), outcome (0.00 ± 14.80) | Baseline (80.00 ± 15.00), outcome (-10.00 ± 17.70) | Baseline (80.00 ± 10.00), outcome (0.00 ± 10.00) |
| Sathyapalan et al. 2017 [22] | Baseline (NR ± NR), outcome (-2.32 ± 15.84) | Baseline (NR ± NR), outcome (-4.10 ± 15.05) | Baseline (NR ± NR), outcome (-2.47 ± 11.28) | Baseline (NR ± NR), outcome (-1.04 ± 11.46) |

Table S2 (Continued)

| **First author, year** | **SBP (mmHg)** | | **DBP (mmHg)** | |
| --- | --- | --- | --- | --- |
|  | **Soy isoflavone** | **Control** | **Soy isoflavone** | **Control** |
| Amanat et al.  2018 [23] | Baseline (130.38 ± 14.66), outcome (0.08 ± 14.37) | Baseline (127.56 ± 1.23), outcome (-0.48 ± 12.43) | Baseline (89.35 ± 9.60), outcome (2.29± 9.54) | Baseline (88.29 ± 10.03), outcome (1.29 ± 9.74) |
| Sathyapalan et al. 2018 [24] | Baseline (125.00 ± 20.20), outcome (-3.80 ± 18.14) | Baseline (124.60 ± 18.80), outcome (-1.20 ± 17.57) | Baseline (77.00 ± 13.80), outcome (-0.20 ± 12.21) | Baseline (77.20 ± 10.90), outcome (0.20 ± 11.27) |

^a^ All values are expressed as means ± standard deviations. Abbreviation: DBP, diastolic blood pressure; SBP, systolic blood pressure; NR, not report.

Table S3 Assessment of risk of bias of the studies included in the meta-analysis

| **Study citation** | **Random sequence generation** | **Allocation concealment** | **Blinding of participants and personnel** | **Blinding of outcome assessment** | **Incomplete data outcome** | **Selective reporting** | **Other bias** |
| --- | --- | --- | --- | --- | --- | --- | --- |
| Simon et al.  2000 [1] | Low | Low | Low | Low | Low | Low | Unclear |
| Han et al.  2002 [2] | Low | Low | Low | Low | Low | Low | Low |
| Squadrito et al. 2002 [3] | Low | Unclear | Low | Unclear | Low | Low | Low |
| Uesugi et al.  2004 [4] | Low | Unclear | Low | High | Low | Low | Low |
| Colacurci et al. 2005 [5] | Low | Unclear | Low | Low | Low | Low | Low |
| Yildiz et al.  2005 [6] | Low | Unclear | Low | Low | Low | Low | Low |
| Hallund et al.  2006 [7] | Low | Unclear | Low | Low | Low | Low | Unclear |
| Gonzalez et al. 2007 [8] | Low | Low | Low | Low | Low | Low | Low |
| Katz et al.  2007 [9] | Low | Unclear | Low | Low | Low | Low | Low |
| Aubertin-Leheudr et al. 2008 [10] | Low | Low | Low | Low | Low | Low | Unclear |
| Khaodhiar et al. 2008 [11] | Low | Low | Low | Low | High | Low | Unclear |
| Gleason et al.  2009 [12] | Low | Low | Low | Low | Low | Low | Low |
| Llaneza et al.  2010 [13] | Low | Unclear | Low | Unclear | Low | Low | Unclear |
| Wong et al.  2012 [14] | Low | Unclear | Low | Low | Low | Low | Low |
| Chilibeck et al. 2013 [15] | Low | Unclear | Low | Low | Low | Low | Low |
| Irace et al.  2013 [16] | Low | Low | Low | Low | Low | Low | Low |
| Kim et al.  2013 [17] | Low | Low | Low | High | Low | Low | Low |
| Liu et al.  2013 [18] | Low | Unclear | Low | Low | Low | Low | Unclear |
| Squadrito et al. 2013 [19] | Low | Low | Low | Low | Low | Low | Low |
| Cheng et al.  2014 [20] | Low | Unclear | Low | Low | Unclear | Low | Low |
| De Gregorio et al. 2017 [21] | Low | Low | Low | Low | Low | Low | Unclear |
| Sathyapalan et al. 2017 [22] | Low | Low | Low | Low | Low | Low | Low |
| Amanat et al. 2018 [23] | Low | Low | Low | Low | Low | Low | Low |
| Sathyapalan et al. 2018 [24] | Low | Low | Low | Low | Low | Low | Low |

Table S4 Meta regression analyses of outcomes in soy isoflavone dosage and intervention duration

| **Outcomes** | **No of studies** | **Coefficient.** | **SE** | **t-value** | ***P*-value** | **95% CI** |
| --- | --- | --- | --- | --- | --- | --- |
| Soy isoflavone dosage | | | | | | |
| SBP (mmHg) | 26 | 0.01 | 0.01 | 0.67 | 0.51 | (-0.01, 0.03) |
| DBP (mmHg) | 26 | 0.00 | 0.06 | 0.21 | 0.83 | (-0.01, 0.02) |
| Intervention duration | | | | | | |
| SBP (mmHg) | 26 | -0.05 | 0.10 | -0.46 | 0.65 | (-0.26, 0.17) |
| DBP (mmHg) | 26 | 0.04 | 0.06 | 0.61 | 0.55 | (-0.09, 0.16) |

Abbreviation: DBP, diastolic blood pressure; SBP, systolic blood pressure; SE, standard error; 95% CI, 95% confidence interval.

**References**

1. Simons LA, von Konigsmark M, Simons J, Celermajer DS. Phytoestrogens do not influence lipoprotein levels or endothelial function in healthy, postmenopausal women. Am J Cardiol. 2000;85:1297-1301.

2. Han KK, Soares JM Jr, Haidar MA, de Lima GR, Baracat EC. Benefits of soy isoflavone therapeutic regimen on menopausal symptoms. Obstet Gynecol. 2002;99:389-394.

3. Squadrito F, Altavilla D, Morabito N, Crisafulli A, D'Anna R, Corrado F, et al. The effect of the phytoestrogen genistein on plasma nitric oxide concentrations, endothelin-1 levels and endothelium dependent vasodilation in postmenopausal women. Atherosclerosis. 2002;163:339-347.

4. Uesugi S, Watanabe S, Ishiwata N, Uehara M, Ouchi K. Effects of isoflavone supplements on bone metabolic markers and climacteric symptoms in Japanese women. Biofactors. 2004;22:221-228.

5. Colacurci N, Chiàntera A, Fornaro F, de Novellis V, Manzella D, Arciello A, et al. Effects of soy isoflavones on endothelial function in healthy postmenopausal women. Menopause. 2005;12:299-307.

6. Yildiz MF, Kumru S, Godekmerdan A, Kutlu S. Effects of raloxifene, hormone therapy, and soy isoflavone on serum high-sensitive C-reactive protein in postmenopausal women. Int J Gynaecol Obstet. 2005;90:128-133.

7. Hallund J, Bügel S, Tholstrup T, Ferrari M, Talbot D, Hall WL, et al. Soya isoflavone-enriched cereal bars affect markers of endothelial function in postmenopausal women. Br J Nutr. 2006;95:1120-1126.

8. González S, Jayagopal V, Kilpatrick ES, Chapman T, Atkin SL. Effects of isoflavone dietary supplementation on cardiovascular risk factors in type 2 diabetes. Diabetes Care. 2007;30:1871-1873.

9. Katz DL, Evans MA, Njike VY, Hoxley ML, Nawaz H, Comerford BP, Sarrel PM. Raloxifene, soy phytoestrogens and endothelial function in postmenopausal women. Climacteric. 2007;10:500-507.

10. Aubertin-Leheudre M, Lord C, Khalil A, Dionne IJ. Isoflavones and clinical cardiovascular risk factors in obese postmenopausal women: a randomized double-blind placebo-controlled trial. J Womens Health (Larchmt). 2008;17:1363-1369.

11. Khaodhiar L, Ricciotti HA, Li L, Pan W, Schickel M, Zhou J, et al. Daidzein-rich isoflavone aglycones are potentially effective in reducing hot flashes in menopausal women. Menopause. 2008;15:125-132.

12. Gleason CE, Carlsson CM, Barnet JH, Meade SA, Setchell KD, Atwood CS, et al. A preliminary study of the safety, feasibility and cognitive efficacy of soy isoflavone supplements in older men and women. Age Ageing. 2009;38:86-93.

13. Llaneza P, Gonzalez C, Fernandez-Iñarrea J, Alonso A, Diaz-Fernandez MJ, Arnott I, et al. Soy isoflavones, Mediterranean diet, and physical exercise in postmenopausal women with insulin resistance. Menopause. 2010;17:372-378.

14. Wong WW, Taylor AA, Smith EO, Barnes S, Hachey DL. Effect of soy isoflavone supplementation on nitric oxide metabolism and blood pressure in menopausal women. Am J Clin Nutr. 2012;95:1487-1494.

15. Chilibeck PD, Vatanparast H, Pierson R, Case A, Olatunbosun O, Whiting SJ, et al. Effect of exercise training combined with isoflavone supplementation on bone and lipids in postmenopausal women: a randomized clinical trial. J Bone Miner Res. 2013;28:780-793.

16. Irace C, Marini H, Bitto A, Altavilla D, Polito F, Adamo EB, et al. Genistein and endothelial function in postmenopausal women with metabolic syndrome. Eur J Clin Invest. 2013;43:1025-1031.

17. Kim J, Lee H, Lee O, Lee KH, Lee YB, Young KD, et al. Isoflavone supplementation influenced levels of triglyceride and luteunizing hormone in Korean postmenopausal women. Arch Pharm Res. 2013;36:306-313.

18. Liu ZM, Ho SC, Chen YM, Woo J. Effect of soy protein and isoflavones on blood pressure and endothelial cytokines: a 6-month randomized controlled trial among postmenopausal women. J Hypertens. 2013;31:384-392.

19. Squadrito F, Marini H, Bitto A, Altavilla D, Polito F, Adamo EB, et al. Genistein in the metabolic syndrome: results of a randomized clinical trial. J Clin Endocrinol Metab. 2013;98:3366-3374.

20. Cheng WC, Lo SC, Tsai KS, Tu ST, Wu JS, Chang CI, et al. Effects of high-dose phytoestrogens on circulating cellular microparticles and coagulation function in postmenopausal women. J Formos Med Assoc. 2015;114:710-716.

21. De Gregorio C, Marini H, Alibrandi A, Di Benedetto A, Bitto A, Adamo EB, et al. Genistein supplementation and cardiac function in postmenopausal women with metabolic syndrome: results from a pilot strain-echo study. Nutrients. 2017;9:584.

22. Sathyapalan T, Rigby AS, Bhasin S, Thatcher NJ, Kilpatrick ES, Atkin SL. Effect of soy in men with type 2 diabetes mellitus and subclinical hypogonadism: a randomized controlled study. J Clin Endocrinol Metab. 2017;102:425-433.

23. Amanat S, Eftekhari MH, Fararouei M, Bagheri Lankarani K, Massoumi SJ. Genistein supplementation improves insulin resistance and inflammatory state in non-alcoholic fatty liver patients: a randomized, controlled trial. Clin Nutr. 2018;37:1210-1215.

24. Sathyapalan T, Aye M, Rigby AS, Thatcher NJ, Dargham SR, Kilpatrick ES, et al. Soy isoflavones improve cardiovascular disease risk markers in women during the early menopause. Nutr Metab Cardiovasc Dis. 2018;28(7):691-697.
