# Supplementary material for: Effect of soy isoflavone supplementation on blood pressure: a meta-analysis of randomized controlled trials
Source: Nutr J. 2024 Mar 7;23:32. doi: 10.1186/s12937-024-00932-6 (PMC10918941; doi:10.1186/s12937-024-00932-6)
Supplement: Supplementary file 2 — Supplementary Material 2. [file 12937_2024_932_MOESM2_ESM.pdf]

Fig. S1A

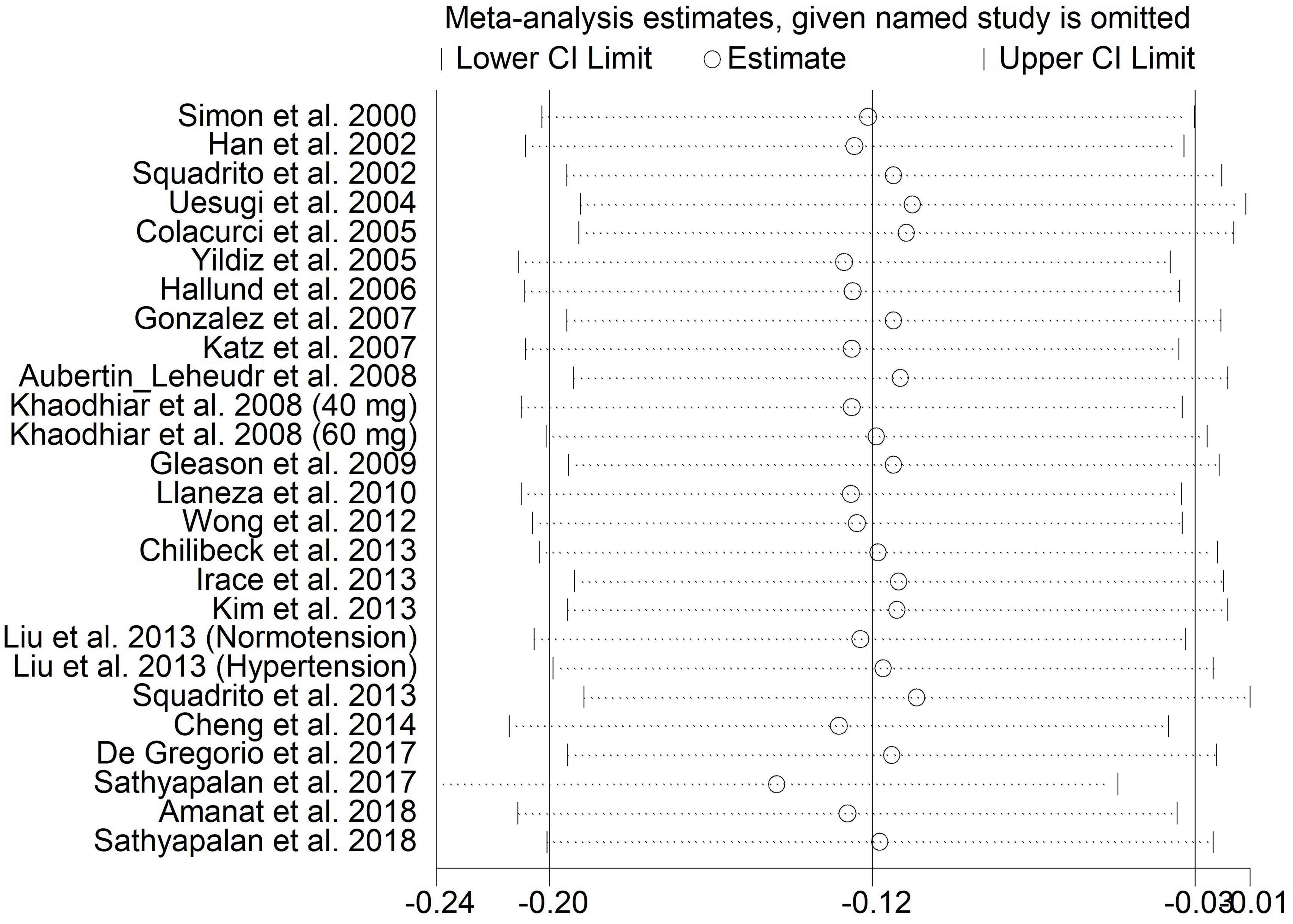
Meta-analysis estimates, given named study is omitted
Lower CI Limit
Estimate
Upper CI Limit
Simon et al. 2000
Han et al. 2002
Squadrito et al. 2002
Uesugi et al. 2004
Colacurci et al. 2005
Yildiz et al. 2005
Hallund et al. 2006
Gonzalez et al. 2007
Katz et al. 2007
Aubertin_Leheudr et al. 2008
Khaodhiar et al. 2008 (40 mg)
Khaodhiar et al. 2008 (60 mg)
Gleason et al. 2009
Llaneza et al. 2010
Wong et al. 2012
Chilibeck et al. 2013
Irace et al. 2013
Kim et al. 2013
Liu et al. 2013 (Normotension)
Liu et al. 2013 (Hypertension)
Squadrito et al. 2013
Cheng et al. 2014
De Gregorio et al. 2017
Sathyapalan et al. 2017
Amanat et al. 2018
Sathyapalan et al. 2018
-0.24
-0.20
-0.12
-0.03
0.01

Fig. S1B

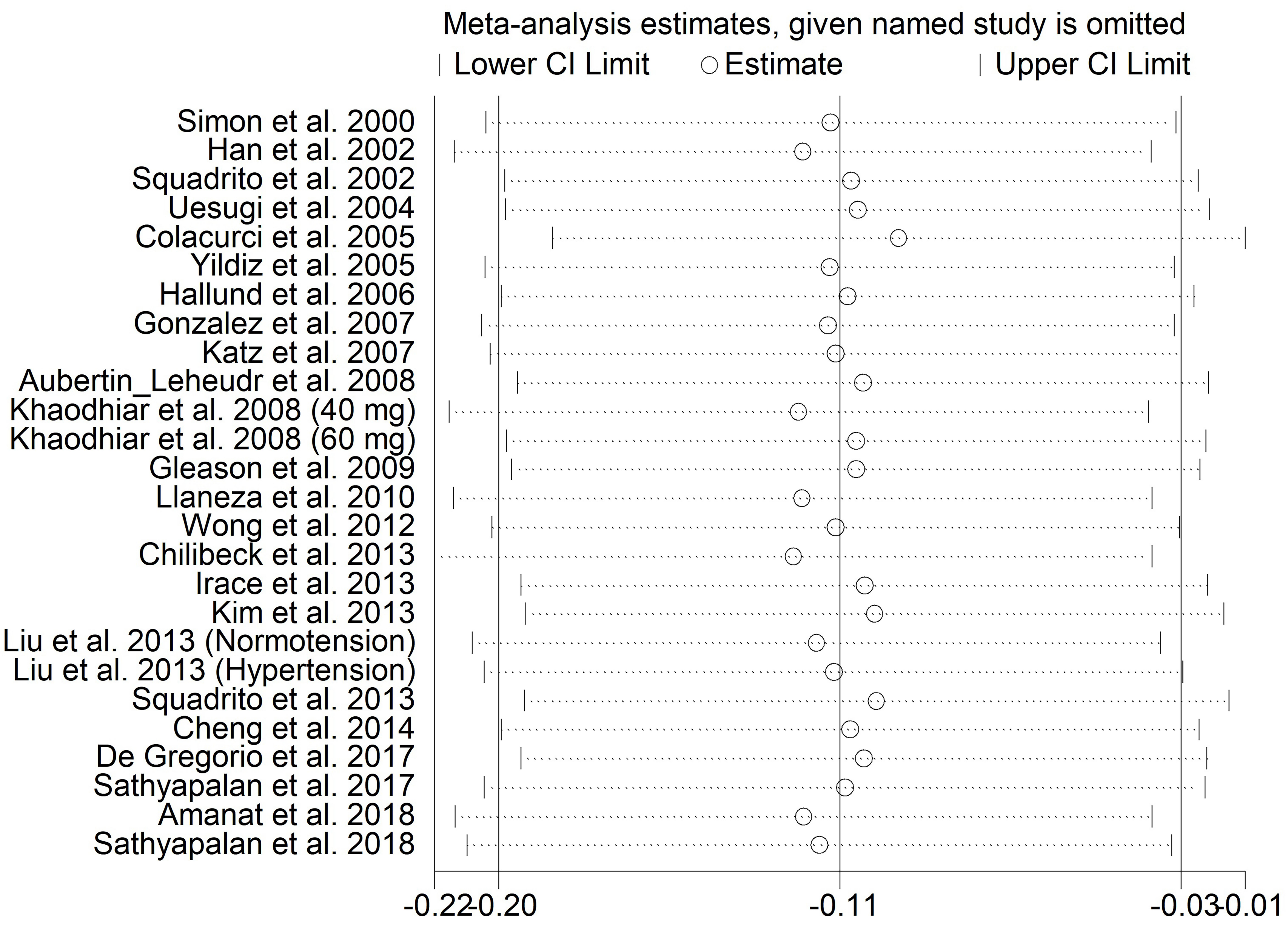

Meta-analysis estimates, given named study is omitted
Lower CI Limit
Estimate
Upper CI Limit
Simon et al. 2000
Han et al. 2002
Squadrito et al. 2002
Uesugi et al. 2004
Colacurci et al. 2005
Yildiz et al. 2005
Hallund et al. 2006
Gonzalez et al. 2007
Katz et al. 2007
Aubertin_Leheudr et al. 2008
Khaodhiar et al. 2008 (40 mg)
Khaodhiar et al. 2008 (60 mg)
Gleason et al. 2009
Llaneza et al. 2010
Wong et al. 2012
Chilibeck et al. 2013
Irace et al. 2013
Kim et al. 2013
Liu et al. 2013 (Normotension)
Liu et al. 2013 (Hypertension)
Squadrito et al. 2013
Cheng et al. 2014
De Gregorio et al. 2017
Sathyapalan et al. 2017
Amanat et al. 2018
Sathyapalan et al. 2018
-0.22
-0.20
-0.11
-0.03
-0.01

Fig. S2A

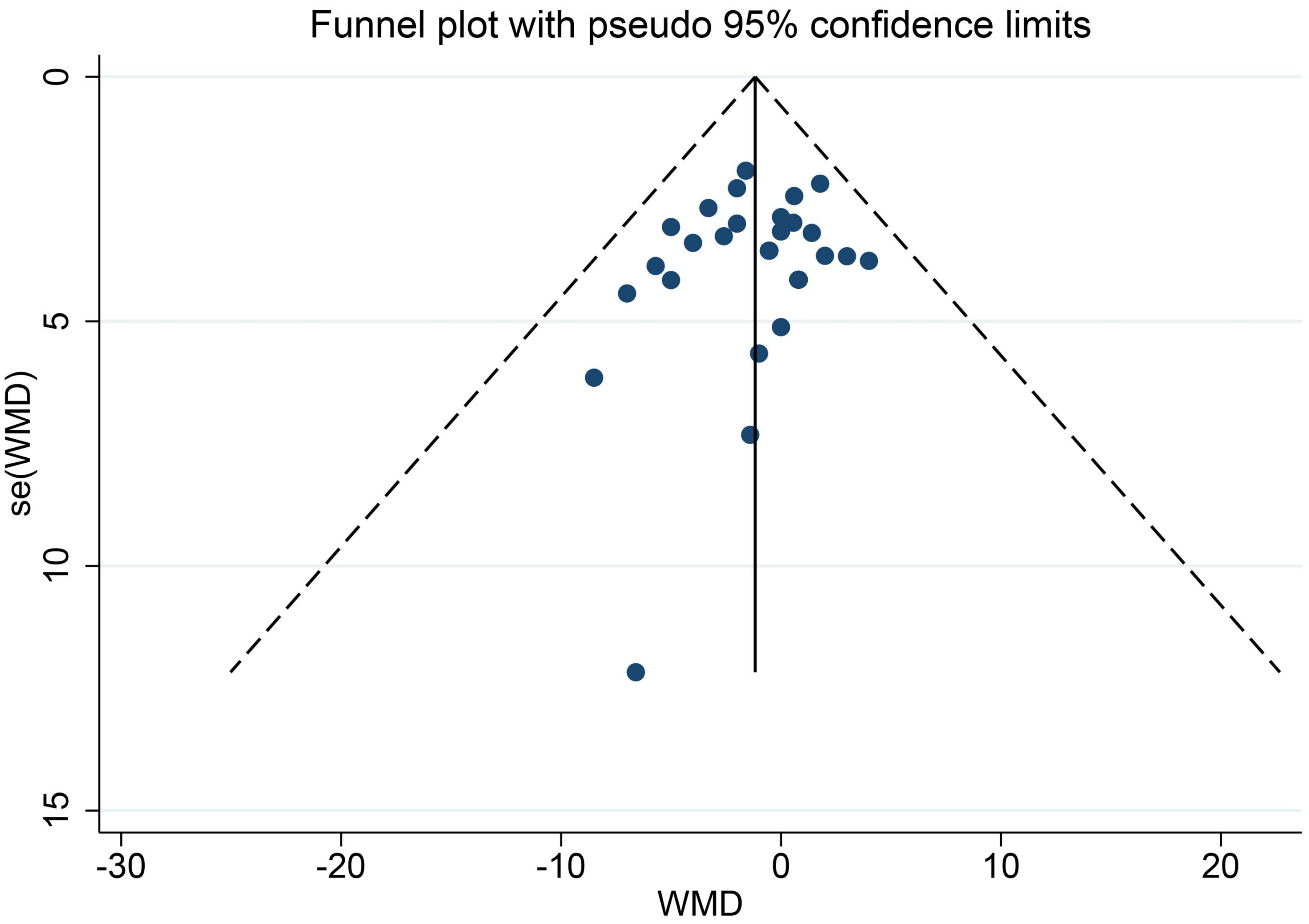

Funnel plot with pseudo 95% confidence limits
0
5
10
15
se(WMD)
-30
-20
-10
0
10
20
WMD

Fig. S2B

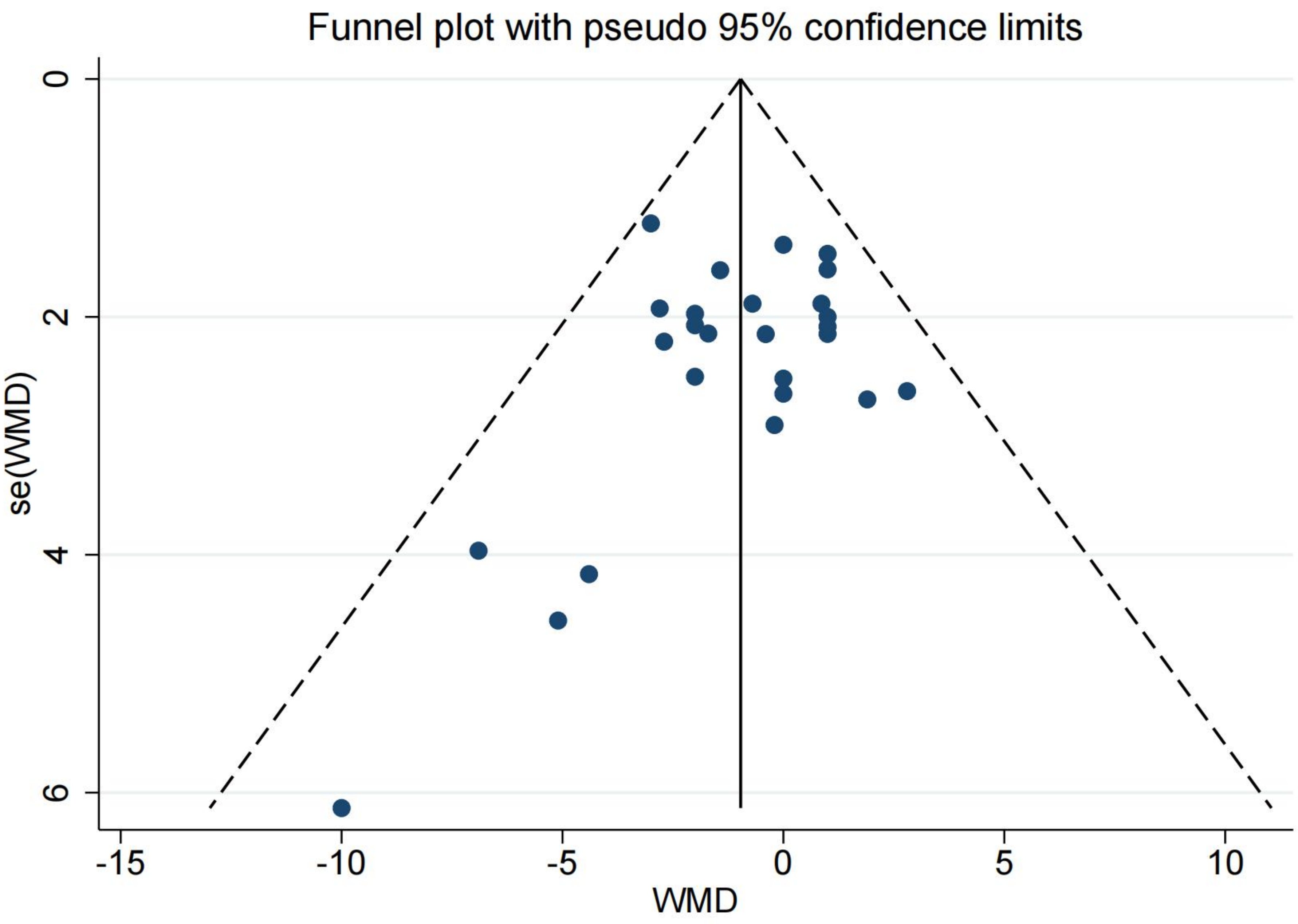

Funnel plot with pseudo 95% confidence limits
se(WMD)
0
2
4
6
-15
-10
-5
0
5
10
WMD

Supplemental Figure Legends

Fig. S1A Sensitivity analyses for the effect of soy isoflavone supplementation on SBP. Abbreviation: SBP, systolic blood pressure.

Fig. S1B Sensitivity analyses for the effect of soy isoflavone supplementation on DBP. Abbreviation: DBP, diastolic blood pressure.

Fig. S2A Funnel plot for the effect of soy isoflavone supplementation on SBP. Abbreviation: SBP, systolic blood pressure.

Fig. S2B Funnel plot for the effect of soy isoflavone supplementation on DBP. Abbreviation: DBP, diastolic blood pressure.
