# Supplementary material for: Effect of soy isoflavone supplementation on blood pressure: a meta-analysis of randomized controlled trials
Source: Nutr J. 2024 Mar 7;23:32. doi: 10.1186/s12937-024-00932-6 (PMC10918941; doi:10.1186/s12937-024-00932-6)
Supplement: Supplementary file 3 — Supplementary Material 3. [file 12937_2024_932_MOESM3_ESM.doc]

Table 1 Characteristics of twenty-four randomized controlled trials included in the meta-analysis

| **First author, year** | **Country** | **Duration of intervention**  **(months)** | **Study design** | **Sample size (isoflavone/**  **control)** | **Mean age (year)** | **BMI (kg/m^2^)** | **Women (%)** | **Population** | **Intervention** | |
| --- | --- | --- | --- | --- | --- | --- | --- | --- | --- | --- |
|  |  |  |  |  |  |  |  |  | **Soy isoflavone group** | **Control group** |
| Simon et al.  2000 [42] | Australia | 2 | Crossover | 20/20 | 59.0 | 26.8 | 100.0 | Healthy postmenopausal women | 80 mg soy isoflavone  (PhytoLife 1) | Placebo (NR) |
| Han et al.  2002 [43] | Brazil | 4 | Parallel | 40/40 | 48.5 | 24.9 | 100.0 | Postmenopausal women | 100 mg soy isoflavone (50.3 mg soy protein and 33.3 mg isoflavone per capsule) | Placebo (50.3 mg soy protein and 33.3 mg glucose per capsule) |
| Squadrito et al. 2002 [44] | Italy | 6 | Parallel | 30/30 | 56.0 | NR | 100.0 | Healthy  Postmenopausal women | 54 mg soy isoflavone (genistein) | Placebo (NR) |
| Uesugi et al.  2004 [45] | Japan | 1 | Crossover | 58/58 | 58.0 | 23.0 | 100.0 | Climacteric women | 40 mg soy isoflavone (3.4 mg daidzein, 0.9 mg genistein, and 2.7 mg glycitein per gram of tablet) | Placebo (same matrix without isoflavone) |
| Colacurci et al. 2005 [24] | Italy | 6 | Parallel | 29/28 | 55.2 | 25.9 | 100.0 | Healthy postmenopausal women | 120 mg soy isoflavone (60 mg genistein and 60 mg daidzein) | Placebo (NR) |
| Yildiz et al.  2005 [25] | Turkey | 6 | Parallel | 20/20 | 50.0 | 27.2 | 100.0 | Healthy postmenopausal women | 40 mg soy isoflavone (genistein) | Placebo (NR) |
| Hallund et al.  2006 [46] | Denmark | 2 | Crossover | 30/28 | 57.0 | 24.0 | 100.0 | Healthy postmenopausal women | Cereal bars with 50 mg soy isoflavone (genistein:daidzein ratio of 2:1) | Placebo (cereal bars) |
| Gonzalez et al. 2007 [47] | United Kingdom | 3 | Crossover | 26/26 | NR | 31.0 | 100.0 | Postmenopausal women with type 2 diabetes | 132 mg soy isoflavone (53% genistein, 37% daidzein, and 10% glycitein) | Placebo (microcrystalline cellulose) |
| Katz et al.  2007 [48] | United States | 1.5 | Crossover | 22/22 | 58.5 | 27.6 | 100.0 | Healthy postmenopausal women | 55 mg soy isoflavone (daidzein and genistein) | Placebo (NR) |
| Aubertin-Leheudr et al. 2008 [49] | Canada | 6 | Parallel | 25/25 | 57.4 | 32.0 | 100.0 | Postmenopausal obese women | 70 mg soy isoflavone  (44 mg daidzein, 16 mg glycitein, and 10 mg genistein) | Placebo (NR) |
| Khaodhiar et al. 2008 [50] | United States | 3 | Parallel | 97/45 | 53.1 | 28.6 | 100.0 | Healthy  menopausal women | 40 or 60 mg soy isoflavone (70% daidzein, 10% genistein, and 20% glycitein) | Placebo (NR) |
| Gleason et al. 2009 [51] | United States | 6 | Parallel | 15/15 | 73.7 | NR | 50.0 | Healthy men and postmenopausal women | 100 mg soy isoflavone (85% daidzein and genistein) | Placebo (maltodextrin and caramel food colour) |

Table 1 (Continued)

| **First author, year** | **Country** | **Duration of intervention**  **(months)** | **Study design** | **Sample size (isoflavone/**  **control)** | **Mean age (year)** | **BMI (kg/m^2^)** | **Women (%)** | **Population** | **Intervention** | |
| --- | --- | --- | --- | --- | --- | --- | --- | --- | --- | --- |
|  |  |  |  |  |  |  |  |  | **Soy isoflavone group** | **Control group** |
| Llaneza et al.  2010 [52] | Spain | 24 | Parallel | 58/58 | 56.4 | 30.0 | 100.0 | Postmenopausal women with insulin resistance | Mediterranean diet with 40 mg soy isoflavone (NR) | Placebo (mediterranean diet) |
| Wong et al. 2012 [53] | United States | 1.5 | Parallel | 12/12 | 55.7 | 25.4 | 100.0 | Postmenopausal women | 80 mg soy isoflavone (22.0 mg daidzein, 13.5 mg glycitein, and 5.0 mg genistein per tablet) | Placebo (1.0 mg aglycone equivalent per tablet) |
| Chilibeck et al. 2013 [54] | Canada | 24 | Parallel | 90/88 | 56.6 | 27.1 | 100.0 | Postmenopausal women | 165 mg soy isoflavone (105 mg aglycone equivalent) | Placebo (dicalcium phosphate, magnesium stearate, and sorbitol) |
| Irace et al.  2013 [26] | Italy | 6 | Parallel | 10/10 | 58.8 | 31.8 | 100.0 | Postmenopausal women with metabolic syndrome | 54 mg soy isoflavone (genistein) | Placebo (NR) |
| Kim et al.  2013 [55] | Korea | 3 | Parallel | 42/43 | 53.6 | 23.2 | 100.0 | Postmenopausal women with type 2 diabetes | 70 mg soy isoflavone (38.0 mg glycitein, 20 mg daidzein, and 12.4 mg genistein) | Placebo (NR) |
| Liu et al.  2013 [21] | China | 6.25 | Parallel | 60/60 | NR | NR | 100.0 | Postmenopausal women | 15 g milk protein and 100 mg  isoflavone (35 mg daidzein, 59 mg genistein, and 4 mg glycitein) | Placebo (15 g milk protein) |
| Squadrito et al. 2013 [22] | Italy | 12 | Parallel | 60/60 | 55.5 | 31.8 | 100.0 | Postmenopausal women with metabolic syndrome | 54 mg soy isoflavone (genistein) | Placebo (NR) |
| Cheng et al. 2015 [56] | China | 12 | Parallel | 41/41 | 56.6 | 23.0 | 100.0 | Postmenopausal women | 300 mg isoflavone aglycone (NR) | Placebo (NR) |
| De Gregorio et al. 2017 [57] | Italy | 12 | Parallel | 11/11 | NR | 30.3 | 100.0 | Postmenopausal women with metabolic syndrome | 54 mg soy isoflavone (genistein) | Placebo (NR) |
| Sathyapalan et al. 2017 [58] | United Kingdom | 3 | Parallel | 100/100 | 52.0 | 31.7 | 0.0 | Men with type 2 diabetes | 15 g soy protein with 66 mg isoflavone (54% genistein, 35% daidzein, and 11% glycitein) | Placebo (15 g soy protein) |

Table 1 (Continued)

| **First author, year** | **Country** | **Duration of intervention**  **(months)** | **Study design** | **Sample size (isoflavone/**  **control)** | **Mean age (year)** | **BMI (kg/m^2^)** | **Women (%)** | **Population** | **Intervention** | |
| --- | --- | --- | --- | --- | --- | --- | --- | --- | --- | --- |
|  |  |  |  |  |  |  |  |  | **Soy isoflavone group** | **Control group** |
| Amanat et al. 2018 [59] | Iran | 2 | Parallel | 41/41 | 43.6 | 28.5 | 24.4 | Patients with non-alcoholic fatty liver disease | 250 mg soy isoflavone (genistein) | Placebo (cornstarch) |
| Sathyapalan et al. 2018 [23] | United Kingdom | 6 | Parallel | 60/60 | 52.0 | 25.5 | 100.0 | Early menopause women | 15 g soy protein with 66 mg isoflavone (54% genistein, 35% daidzein, and 11% glycitein) | Placebo (15 g soy protein) |

Abbreviation: BMI, body mass index; NR, not report.

Table 2 Assessment of quality of evidence for outcomes using the GRADE

|  | **Quality assessment** | | | | | | | |  | **No. of patients** | |  | **Quality of**  **evidence**  **(GRADE)** |
| --- | --- | --- | --- | --- | --- | --- | --- | --- | --- | --- | --- | --- | --- |
| **Outcomes** | | **No of studies** | **Study design** | **Risk of bias** | **Imprecision** | **Inconsistency** | **Indirectness** | **Publication bias** |  | **Intervention** | **Control** | **WMD (95%CI)** |
| SBP | 26 | | Randomized  trials | No serious risk  of bias ^a^ | No serious  imprecision ^b^ | No serious  inconsistency ^c^ | Serious  indirectness ^d^ | No serious limitation ^e^ |  | 1005 | 940 | -1.40 (-2.65, -0.14) | ⨁⨁⨁◯  Moderate |
| DBP | 26 | | Randomized  trials | No serious risk  of bias | No serious  imprecision | No serious  inconsistency | Serious  indirectness | No serious limitation |  | 1005 | 940 | -1.11 (-1.91, -0.30) | ⨁⨁⨁◯  Moderate |

The quality of evidence was evaluated at 4 levels using GRADE (high, moderate, low, very low). ^a^ No downgrade for risk of bias, as most studies were assessed as low risk of bias. ^b^ No downgrade for imprecision, as the optimal information size was met and the 95%CI did not include the null value. ^c^ No downgrade for inconsistency, as there was a low of heterogeneity (SBP: *I*^2^ =0.0%, *P*=0.61; DBP: *I*^2^ = 0.0%, *P*=0.87). ^d^ Downgraded for indirectness, as the intake of sodium and potassium was not provided, which may have confounding effects. ^e^ No downgrade for publication bias, as there was no publication bias evaluated by the funnel plots and Egger's test. Abbreviation: DBP, diastolic blood pressure; SBP, systolic blood pressure; GRADE, Grading of Recommendations Assessment, Development and Evaluation; WMD, weighted mean difference; 95% CI, 95% confidence interval.

Table 3 Subgroup analyses of soy isoflavone supplementation on blood pressure in adults

| **Subgroup** |  | **SBP** | | | |  | **DBP** | | | |
| --- | --- | --- | --- | --- | --- | --- | --- | --- | --- | --- |
|  |  |  |  | **Test of heterogeneity** | |  |  |  | **Test of heterogeneity** | |
|  | **No. of studies** | **Net change**  **(95% CI)** | ***P* ^a^** | ***I*^2^ (%)** | ***P* ^b^** | **No. of studies** | **Net change**  **(95% CI)** | ***P* ^a^** | ***I*^2^ (%)** | ***P* ^b^** |
| **Resting blood pressure status** |  |  |  |  |  |  |  |  |  |  |
| Normotension | 6 | -0.77 (-3.21, 1.68) | 0.54 | 0.00 | 0.73 | 6 | -0.95 (-2.55, 1.77) | 0.24 | 0.00 | 0.68 |
| Prehypertension | 16 | -1.99 (-3.64, -0.35) | 0.02 | 0.00 | 0.60 | 16 | -1.05 (-2.08, -0.02) | 0.04 | 0.00 | 0.59 |
| Hypertension | 2 | -1.65 (-7.18, 3.89) | 0.56 | 59.00 | 0.12 | 2 | -1.43 (-4.63, 1.77) | 0.38 | 0.00 | 0.61 |
| **Dose of soy isoflavone (mg/day)** |  |  |  |  |  |  |  |  |  |  |
| ＜50 | 3 | 1.11 (-2.74, 4.95) | 0.57 | 0.00 | 0.81 | 3 | -0.24 (-2.31, 1.82) | 0.81 | 0.00 | 0.72 |
| ≥50, ≤100 | 18 | -1.67 (-3.22, -0.12) | 0.03 | 9.10 | 0.46 | 18 | -1.28 (-2.36, -0.19) | 0.02 | 0.00 | 0.82 |
| ＞100 | 5 | -1.77 (-4.37, 0.83) | 0.18 | 0.00 | 0.51 | 5 | -1.23 (-2.69, 0.24) | 0.10 | 6.40 | 0.37 |
| **Duration of follow-up**  **(months)** |  |  |  |  |  |  |  |  |  |  |
| ＜6 | 12 | -0.61 (-2.46, 1.24) | 0.52 | 0.00 | 0.82 | 12 | -0.75 (-1.91, 0.40) | 0.20 | 0.00 | 0.95 |
| ≥6 | 14 | -2.08 (-3.80, -0.36) | 0.02 | 10.30 | 0.34 | 14 | -1.43 (-2.54, -0.32) | 0.01 | 0.00 | 0.51 |
| **Types of soy isoflavone** |  |  |  |  |  |  |  |  |  |  |
| Genistein | 6 | -2.29 (-5.71, 1.14) | 0.19 | 46.20 | 0.10 | 6 | -1.59 (-3.86, 0.68) | 0.17 | 25.30 | 0.24 |
| Mixed types of soy isoflavone | 19 | -1.39 (-2.78, -0.01) | 0.04 | 0.00 | 0.84 | 19 | -1.00 (-1.89, -0.13) | 0.02 | 0.00 | 0.92 |
| **Health status** |  |  |  |  |  |  |  |  |  |  |
| Health | 16 | -1.68 (-3.33, -0.04) | 0.04 | 0.00 | 0.63 | 16 | -1.07 (-2.04, -0.10) | 0.03 | 0.00 | 0.88 |
| Diabetes | 3 | -0.92 (-4.00, 2.15) | 0.57 | 39.00 | 0.19 | 3 | -1.61 (-3.78, 0.57) | 0.15 | 0.00 | 0.67 |
| Metabolic syndrome | 3 | -12.60 (-20.98, -4.21) | <0.01 | 0.00 | 0.89 | 3 | -6.55 (-11.33, -1.78) | 0.01 | 0.00 | 0.78 |
| Hypertension | 2 | -1.65 (-7.18, 3.89) | 0.56 | 59.00 | 0.12 | 2 | -1.43 (-4.63, 1.77) | 0.38 | 0.00 | 0.61 |
| Non-alcoholic fatty  liver disease | 1 | 0.56 (-5.29, 6.41) | 0.85 |  |  | 1 | 1.00 (-3.20, 5.20) | 0.64 |  |  |
| Insulin resistance | 1 | 0.10 (-6.57, 6.77) | 0.98 |  |  | 1 | 0.80 (-3.05, 4.65) | 0.68 |  |  |
| **Baseline BMI**  **(kg/m^2^)** |  |  |  |  |  |  |  |  |  |  |
| ＜25 | 5 | -1.04 (-3.67, 1.59) | 0.44 | 0.00 | 0.45 | 5 | -0.99 (-2.84, 0.85) | 0.29 | 0.00 | 0.49 |
| ≥25 | 18 | -1.35 (-2.86, 0.17) | 0.08 | 0.00 | 0.48 | 18 | -0.82 (-1.72, 0.08) | 0.07 | 0.00 | 0.66 |
| **Mean age** |  |  |  |  |  |  |  |  |  |  |
| ＜50 | 2 | 0.30 (-3.96, 4.55) | 0.89 | 0.00 | 0.90 | 2 | 1.00 (-1.87, 3.87) | 0.49 | 0.00 | 1.00 |
| ≥50 | 23 | -1.30 (-2.61, 0.01) | 0.05 | 1.90 | 0.45 | 23 | -1.28 (-2.13, -0.43) | 0.01 | 0.00 | 0.95 |

Table 3 (Continued)

| **Subgroup** |  | **SBP** | | | |  | **DBP** | | | |
| --- | --- | --- | --- | --- | --- | --- | --- | --- | --- | --- |
|  |  |  |  | **Test of heterogeneity** | |  |  |  | **Test of heterogeneity** | |
|  | **No. of studies** | **Net change**  **(95% CI)** | ***P* ^a^** | ***I*^2^ (%)** | ***P* ^b^** | **No. of studies** | **Net change**  **(95% CI)** | ***P* ^a^** | ***I*^2^ (%)** | ***P* ^b^** |
| **Gender** |  |  |  |  |  |  |  |  |  |  |
| Female | 23 | -1.73 (-3.09, -0.37) | 0.01 | 0.00 | 0.68 | 23 | -1.26 (-2.17, -0.34) | 0.01 | 0.00 | 0.80 |
| Male | 1 | 1.78 (-2.50, 3.66) | 0.42 |  |  | 1 | -1.43 (-4.58, 1.72) | 0.37 |  |  |
| Both sexes | 2 | -1.17 (-6.43, 4.10) | 0.66 | 43.00 | 0.16 | 2 | -0.13 (-3.87, 3.60) | 0.95 | 24.80 | 0.25 |

^a^ *P* values for difference between subgroups. ^b^ *P* values for heterogeneity test within subgroups. Abbreviation: BMI, body mass index; DBP, diastolic blood pressure; SBP, systolic blood pressure; 95% CI, 95% confidence interval.
